# Non-adiabatic dynamics close to conical intersections and the surface hopping perspective

**DOI:** 10.3389/fchem.2014.00097

**Published:** 2014-11-21

**Authors:** João Pedro Malhado, Michael J. Bearpark, James T. Hynes

**Affiliations:** ^1^Department of Chemistry, Imperial College LondonUK; ^2^Department of Chemistry and Biochemistry, University of Colorado, BoulderBoulder, CO, USA; ^3^Département de Chimie, École Normale Supérieur, UMR ENS-CNRS-UPMC 8640Paris, France

**Keywords:** non-adiabatic dynamics, conical intersections, surface hopping, Landau-Zener, decoherence, Born-Oppenheimer approximation

## Abstract

Conical intersections play a major role in the current understanding of electronic de-excitation in polyatomic molecules, and thus in the description of photochemistry and photophysics of molecular systems. This article reviews aspects of the basic theory underlying the description of non-adiabatic transitions at conical intersections, with particular emphasis on the important case when the dynamics of the nuclei are treated classically. Within this classical nuclear motion framework, the main aspects of the surface hopping methodology in the conical intersection context are presented. The emerging picture from this treatment is that of electronic transitions around conical intersections dominated by the interplay of the nuclear velocity and the derivative non-adiabatic coupling vector field.

## 1. Introduction

The Born-Oppenheimer or adiabatic approximation (Born and Huang, 1968) for the separation of electronic and nuclear motion is at the heart of any quantum mechanical treatment of chemical phenomena. It is a requirement in almost all electronic structure methods and its implications are equally important in dynamical studies. Even the most basic notion of molecular structure can only be sustained within this approximation (Woolley and Sutcliffe, 1976; Cafiero and Adamowicz, 2005). As fundamental as it is, there are nonetheless chemical phenomena for which the Born-Oppenheimer approximation does not hold, being dominated by the so called non-adiabatic effects (Yarkony, 2012; Yonehara et al., 2012). As will be discussed in this article, an important case where this approximation breaks down is in the vicinity of conical intersections, where as a consequence, the dynamics is dominated by a strong coupling between nuclear and electronic motion.

Conical intersections play a central role in photochemistry (Michl and Bonačić-Koutecký, 1990; Klessinger, 1995; Bernardi et al., 1996; Domcke et al., 2011), since by definition a photochemical process involves several electronic states of a molecular system, and at conical intersections these states are strongly coupled, thereby promoting efficient electronic transitions. Understanding the dynamics of the system in the vicinity of conical intersections is therefore essential to rationalize and predict the rate and the product distribution of photochemical reactions.

This article aims to provide an overview of the basic theoretical concepts involved in the description of non-adiabatic effects in the dynamics of molecular systems in general, and in the vicinity of conical intersections in particular. The picture resulting from the Born-Oppenheimer approximation is that the atoms' position and dynamics, or that of the nuclei at their center, is determined by a potential energy surface generated by the electrons. Such picture lends itself to a description of the dynamics according to classical mechanics, which has been exceedingly successful in the description of chemical phenomena of atoms heavier than helium. The appeal of classical mechanics lays not only in the computational convenience and scalability allowing to study complex and large systems at the molecular scale, but also in its conceptual simplicity providing insight into the mechanism and factors governing chemical change. When the Born-Oppenheimer approximation is not valid the convenient picture of a single potential energy surface is lost, and the electronic and nuclear coupling may be seen as the need to include multiple potential energy surfaces in the description of the dynamics. It is possible to combine a classical dynamics description of the nuclei with this multi-potential energy surface picture through the so called surface hopping approach (Bjerre and Nikitin, 1967; Tully and Preston, 1971; Tully, 1990; Drukker, 1999; Persico and Granucci, 2014). Surface hopping methods have proved to be an invaluable tool to study photochemistry, providing insight and a mechanistic picture for the outcome of these reactions. See for example reference (Bearpark et al., 1996), whose results have recently been confirmed by quantum dynamical calculations (Mendive-Tapia et al., 2012), and the study of Garavelli, Cerullo and co-workers, who combined surface hopping calculations and experiment to follow the primary photochemical event in vision (Polli et al., 2010).

This work reviews and compares the description of non-adiabatic effects at a full quantum level and with a surface hopping treatment. The focus is on dynamics around a generic conical intersection, presenting the underlying theory and illustrating the consequences with numerical simulations. The physical picture of electronic transitions that emerges is dominated by the interplay of the nuclear velocity and the derivative non-adiabatic coupling vector field.

The treatment followed in this review remains formal, using Hilbert space vectors and Dirac notation for the benefit of generality and to make clear the nature of the different quantities involved and how they relate to each other. A somewhat high level of mathematical detail is given in the derivations, and while this may weight the manuscript in terms of number of equations, it is hoped that helps the reader understand the origin of some results that are sometimes obscure in the literature.

The review is organized as follows. The presentation begins with a quantum description of the dynamics in Section 2, where both electrons and nuclei are treated quantum mechanically, by introducing the Born-Huang expansion for the state of the system, from which the concept of nuclear wavefunctions arises and the equations for their time evolution are obtained. Non-adiabatic coupling terms, which couple nuclear and electronic motion, are introduced and the Born-Oppenheimer and adiabatic approximations are discussed. Section 3 discusses the diabatic representation, an important alternative representation of the state of the full system which is useful in a number of practical situations, such as when implementing numerical schemes to describe the dynamics of the system with non-adiabatic effects with nuclei treated quantum mechanically, and is a helpful tool in the derivations in the following sections. Conical intersections are introduced in Section 4, along with their main general characteristics. Section 5 derives the equations for electronic quantum dynamics as a function of an arbitrary classical nuclear coordinate. It is shown how the electron dynamics is affected by nuclear dynamics, not addressing the reverse problem of how the nuclear dynamics is affected by the electronic structure. Section 6 discusses the simple and paradigmatic Landau-Zener model for non-adiabatic transitions and its applicability in the conical intersection context. This model illustrates the main features of non-adiabatic transitions and has seen a wide use in applications in the context of surface hopping schemes discussed in Section 7. In this latter section the surface hopping approach to include electron dynamics effects on the classical dynamics of the nuclei is described, along with the current standard surface hopping implementation, the fewest switches algorithm, with a brief description of the application of the algorithm to the case of a conical intersection. Section 7 ends with a discussion about the origin of the electronic coherence issues affecting mixed quantum classical systems. Section 8 provides some concluding remarks.

## 2. Born-oppenheimer or adiabatic approximation

In the description of most chemical phenomena which do not involve the heavier atoms of the periodic table, when relativistic effects (in particular spin-orbit coupling) do not play a role, the dynamics of electrons and nuclei can in general be described by the Schrödinger equation

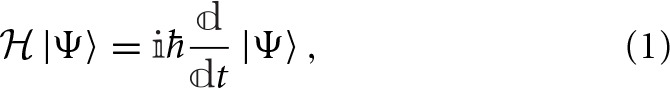



with the Hamiltonian operator

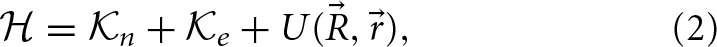



where the first two terms are the kinetic energy of nuclei and electrons, respectively, and the last term is the potential energy of interaction between all the particles, which depends on the positions of the nuclei $\vec{R}$ and electrons $\vec{r}$ defined with respect to the center of mass of the molecular system, but does not explicitly depend on time.

From the point of view of Hamiltonian Equation (2), nuclei and electrons differ essentially in their electric charges which are responsible for the interactions in *U*($\vec{R}$, $\vec{r}$) and in their masses entering the kinetic terms. The difference in mass between these two types of particles is greater than three orders of magnitude, indicating a clear difference in the fundamental time scales of their motion. This intrinsic separation of time scales provides the underlying idea that has been used to address the problem of finding the solutions of Equation (1): the separation of the Hilbert space of these solutions into a tensor product of subspaces associated with the slow nuclear and fast electronic motions (Bohm, 1993)

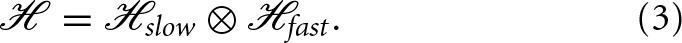



The eigenstates of the nuclear and electronic position operators, |$\vec{R}$〉 and |$\vec{r}$〉, belong to subspaces 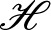
_*slow*_ and 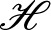
_*fast*_, respectively, and their tensor product |$\vec{R}$〉 ⊗ |$\vec{r}$〉 = |$\vec{R}$, $\vec{r}$〉 is a state in the space 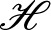.

In the limit of frozen nuclei, the nuclear kinetic energy in Equation (2) is zero, and it is possible to define an electronic Hamiltonian

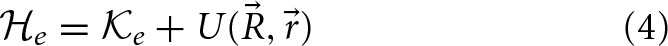



which is defined for each set of positions of the nuclei $\vec{R}$ and where it should be recalled that *U*($\vec{R}$, $\vec{r}$) includes terms representing nuclear repulsions as well as the attractive nuclei-electron and repulsive electron-electron interactions. The eigenvalues *V*_*n*_($\vec{R}$) of the electronic Hamiltonian are thus a function of the nuclear positions, and its eigenstates |ϕ_*n*_; $\vec{R}$〉 also have a parametric dependence on these coordinates

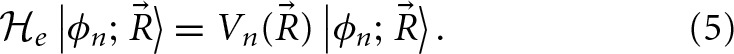



The eigenvalues of the electronic Hamiltonian can also be written as the average electronic energy of its eigenstates:

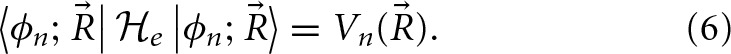



For each value of $\vec{R}$, 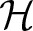
_*e*_ operates on 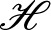
_*fast*_ and its eigenstates can be used as a basis to expand this subspace^1^. The electronic Hamiltonian commutes with the nuclear position operator, [*R*, 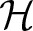
_*e*_] = 0, and thus an arbitrary state |Ψ〉 of the full system may be expanded in terms of a basis of direct products of states |$\vec{R}$〉 ⊗ |ϕ_*n*_; $\vec{R}$〉 = |$\vec{R}$〉, ϕ_*n*_; $\vec{R}$〉,

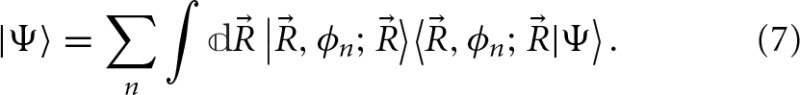



The wavefunction in the coordinate representation (Bohm, 1993) is given by

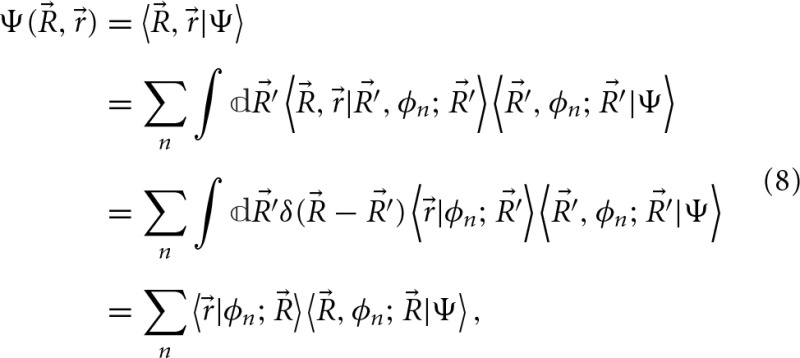



which in conventional wavefunction notation may be written as

(9) $$\Psi (\vec{R},\vec{r})=\sum_{n}\phi_{n}(\vec{R},\vec{r})\chi_{n}(\vec{R}),$$

where the formal definition of the terms is given in Equation (8): the ϕ_*n*_($\vec{R}$, $\vec{r}$) are electronic wavefunctions for clamped nuclei, and are eigenfunctions of Hamiltonian Equation (4). The χ_*n*_($\vec{R}$) are often called nuclear wavefunctions even though these coefficients are not strictly defined in 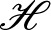
_*slow*_, involving as they do a projection onto a state of 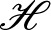
_*fast*_ (the reason for the “nuclear wavefunction” nomenclature will be made more clear presently). The coefficients χ_*n*_($\vec{R}$) = 〈$\vec{R}$, ϕ_*n*_; $\vec{R}$|Ψ〉 carry information about the nuclei position associated with a given electronic eigenstate (labeled by *n*), and their magnitude also quantify the fraction of the total state of the system with electronic state *n* character.

The form Equation (7) of expansion of the state of a molecular system (Born and Huang, 1968) [or the equivalent form Equation (9)] is called the Born-Oppenheimer expansion (or Born-Huang, or adiabatic expansion), and is the starting point for most of the approaches to describe the dynamics of molecular systems.

Many methods exist to calculate the electronic wavefunction of a molecular system for a given nuclear configuration $\vec{R}$ (Helgaker et al., 2000; Levine, 2000). Taking for granted that these wavefunctions can be obtained, along with their eigenvalues *V*_*n*_, the full description of the dynamics of a molecular system still involves the determination of the time evolution of the functions 〈$\vec{R}$, ϕ_*n*_; $\vec{R}$|Ψ〉. This can be done by first taking the scalar product of the Schrödinger Equation (1) with |$\vec{R}$, ϕ_*n*_; $\vec{R}$〉:

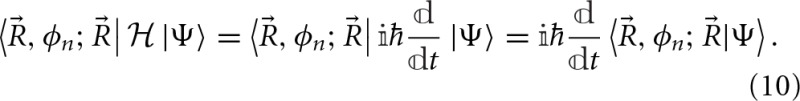



The second equality in Equation (10) is a consequence of the basis states |$\vec{R}$, ϕ_*n*_; $\vec{R}$〉 not depending on time, and indicates that the time evolution of the state of the system can be expressed in terms of the coefficients 〈$\vec{R}$, ϕ_*n*_; $\vec{R}$|Ψ〉 only.

Equations for the time evolution of the coefficients 〈$\vec{R}$, ϕ_*n*_; $\vec{R}$|Ψ〉 can be obtained by rewriting the left hand side of Equation (10) using the Born-Oppenheimer expansion (7). This derivation is done in Appendix Section Equations for the Evolution of Nuclear Wavefuntions, where by choosing a set of rectilinear nuclear coordinates with a diagonal representation of the nuclear kinetic energy operator 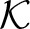
_*n*_ one obtains the equation

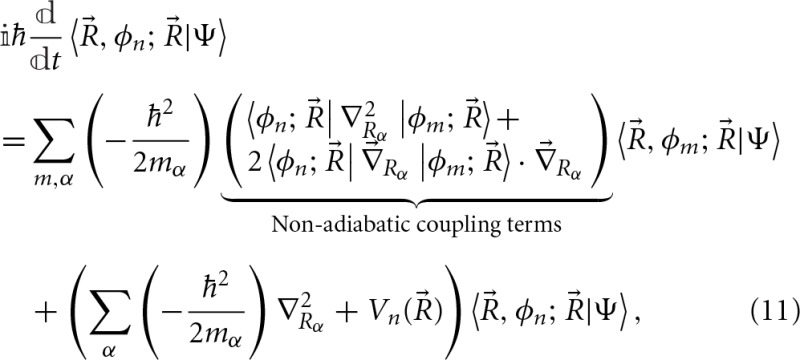



where α and *m*_α_ are, respectively, an index over nuclear coordinates and their associated mass factor. Equation (11) is the fundamental equation that describes the non-adiabatic dynamics of a molecular system. It shows that all terms figuring in the Born-Oppenheimer expansion Equation (9) of the total wavefunction are coupled through nuclei motion, namely by the non-adiabatic coupling terms highlighted in Equation (11). In general, in order to describe the state of the molecule all electronic states must be considered (these are in principle infinite in number, in practice a truncated description suffices).

The Born-Oppenheimer approximation (Born and Huang, 1968) considerably simplifies this picture by neglecting all non-adiabatic coupling terms in Equation (11). This amounts to considering that the electronic eigenstates are very slowly varying functions of the nuclear positions $\vec{R}$; and while they depend on $\vec{R}$, they are independent of the nuclear velocity. This corresponds to the physical picture that the electrons are always equilibrated to the much slower motion of the nuclei. In this case, the Equation (11) are uncoupled and reduce to

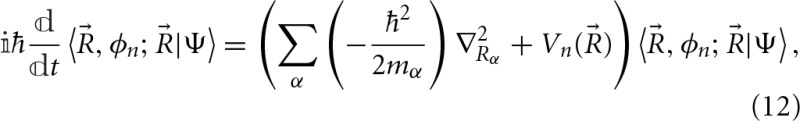



and a system prepared in a given electronic eigenstate |ϕ_*n*_; $\vec{R}$〉 will remain in that same electronic state. As a result, the sum of electronic states in Equation (7) and its equivalent Equation (9) reduces to a single term ^2^

(13) $$\Psi (\vec{R},\vec{r})=\langle \vec{R},\vec{r}|\Psi \rangle =\langle r|\phi_{n};\vec{R}\rangle \langle \vec{R},\phi_{n};\vec{R}|\Psi \rangle =\phi_{n}(\vec{R},\vec{r})\chi_{n}(\vec{R}).$$

Under the Born-Oppenheimer approximation, the system is thus defined in a determined electronic state with the nuclear motion being described by the nuclear wavefunction associated with that state χ_*n*_($\vec{R}$) = 〈$\vec{R}$, ϕ_*n*_; $\vec{R}$|Ψ〉. The evolution of the nuclear wavefunction in Equation (12), by analogy with Equation (1), can be seen to depend on a Hamiltonian formed by the sum of the kinetic energy of the nuclei and the potential energy surface on which the nuclei move, which is equal to the eigenvalues of the electronic Hamiltonian. The idea of the nuclei moving on a potential energy surface generated by the electrons is central to most dynamical treatments in chemistry.

A slightly milder approximation than that of Born-Oppenheimer is to consider that the total state of the system is described by Equation (13) with no approximation on the non-adiabatic coupling terms themselves. In this case Equation (11) reduces to

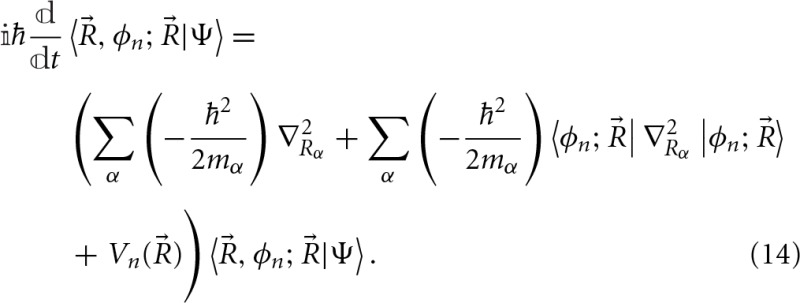



As with the Born-Oppenheimer Equation (12), a given electronic state is uncoupled to the remaining states, but the diagonal non-adiabatic terms ^3^ are retained: they provide for corrections to the potential surfaces for nuclear motion and include the correction of the electronic energies due to the separation of the molecular center of mass motion (Handy and Lee, 1996; Kutzelnigg, 1997). Such corrections depend on the mass of the nuclei, and thus different isotopes will give rise to different potential energy surfaces. This level of approximation is sometimes called adiabatic approximation^4^.

The non-adiabatic coupling terms are not always small and Equation (13) is not always a legitimate description of the total wavefunction of the system, in which case neither of the Equations (12) and (14) give a good account of the dynamics of the system. This is true in particular when the difference in electronic eigenvalues is small (as will be discussed in Section 4, an extreme case of which is provided by conical intersection, where electronic eigenvalues are degenerate). In order to show this, a closer look is given to the non-adiabatic coupling terms in Equation (11). There are two types of coupling: 〈ϕ_*n*_; $\vec{R}$|∇^2^_*R*_|ϕ_*m*_; $\vec{R}$〉 is a scalar quantity termed the scalar or kinetic coupling; 〈ϕ_*n*_; $\vec{R}$|$\vec{\nabla }$_*R*_|ϕ_*m*_; $\vec{R}$〉 is a vectorial quantity in the space of the nuclear coordinates called the vectorial or derivative coupling. By expanding the laplacian operator it is possible to express the kinetic coupling as a function of derivative coupling terms (Domcke et al., 2004)

(15) $$\begin{array}{l} \langle \phi_{n};\vec{R}|\nabla_{R}^{2}|\phi_{m};\;\vec{R}\rangle =\sum_{i}\langle \phi_{n};\vec{R}|{\vec{\nabla }}_{R}|\phi_{i};\;\vec{R}\rangle \langle \phi_{i};\vec{R}|{\vec{\nabla }}_{R}|\phi_{m};\;\vec{R}\rangle \\ \;+\;{\vec{\nabla }}_{R}\cdot \langle \phi_{n};\vec{R}|{\vec{\nabla }}_{R}|\phi_{m};\;\vec{R}\rangle , \end{array}$$

where the second term is the divergence of the derivative coupling vector field. The derivative coupling terms 〈ϕ_*n*_; $\vec{R}$|$\vec{\nabla }$_*R*_|ϕ_*m*_; $\vec{R}$〉 form a anti-hermitian matrix, which implies that for real-valued electronic wavefunctions its diagonal terms are zero [this is the reason why in Equation (14) no derivative couplings appear]. Diagonal terms of the kinetic coupling matrix are not zero due to the first term in Equation (15).

An expression for the derivative coupling off-diagonal elements can be obtained by taking the gradient of the matrix elements of the electronic Hamiltonian in the eigenstate representation and using Equation (5) to find

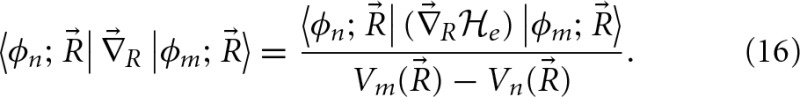



This equation shows that the non-adiabatic coupling terms become important when differences in electronic energy become small, and diverge for nuclear geometries for which electronic states are degenerate. The Born-Oppenheimer (and adiabatic) approximation thus breaks down for degenerate or quasi-degenerate electronic states and the full Equation (11) must be taken into account.

It is important to note that even when dealing with non-adiabatic effects, i.e., when the Born-Oppenheimer or adiabatic approximations are not valid, the concepts of electronic state and of potential energy surfaces are still of value. Crucially, the electronic Hamiltonian Equation (4) is well defined and these surfaces can in principle be calculated ^5^. In such cases, Equation (11) may be seen as describing the evolution of different branches of a nuclear wavefunction evolving in the potential energy surfaces of different electronic eigenstates coupled by non-adiabatic coupling terms. In regions of nuclear space where these terms are important and the magnitude of the coefficients 〈$\vec{R}$, ϕ_*n*_; $\vec{R}$|Ψ〉 in the Born-Oppenheimer expansion Equation (9) vary significantly, the system can be seen as to undertake electronic transitions.

## 3. Diabatic representation

The Born-Oppenheimer expansion Equations (7) or (9) of the state of a molecular system is based on a description that makes use of the eigenstates of the electronic Hamiltonian Equation (5), which are also called adiabatic states. The choice of this basis gives rise to Equation (11) and the non-adiabatic coupling terms within. Equation (16) shows that the non-adiabatic derivative coupling is singular for nuclear configurations where there is a degeneracy of electronic eigenstates, as is the case at conical intersections, discussed in more detail in Section 4. This singularity is problematic in numerical computations when treating the nuclear motion quantum mechanically, and can be circumvented by choosing a different representation for the electronic Hamiltonian, necessarily not diagonal, but for which the derivative coupling is zero. Such a basis is termed a *diabatic* basis.

A diabatic basis can be obtained from the adiabatic one by a unitary transformation

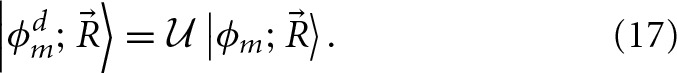



The desired property of the transformation 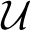 is that in the transformed basis, the derivative coupling between all states and through Equation (15) also the kinetic coupling, is zero (Domcke et al., 2004)

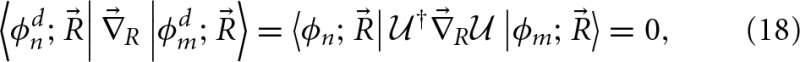



where the gradient operates on both elements to its right. With this result, Equation (11) for the molecular system's state evolution in the diabatic representation is

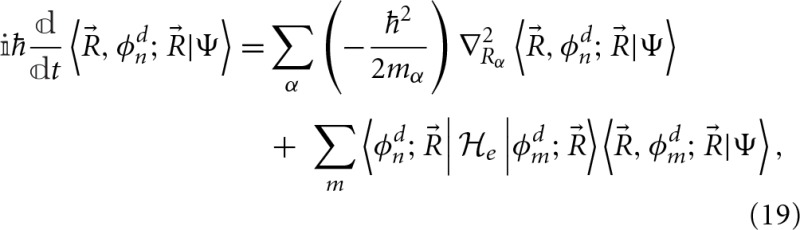



where the second term on the right hand side arises because the diabatic states are *not* eigenstates of the electronic Hamiltonian. Whereas in the adiabatic representation Equation (11) the coupling was due to the nuclear kinetic part, in the diabatic representation Equation (19) the coupling is due to off-diagonal elements of the electronic Hamiltonian. This represents one simplification, since the non-adiabatic derivative coupling was a vectorial quantity, while the present diabatic coupling is a scalar. Further, and more importantly, while derivative couplings are singular at conical intersections, diabatic couplings in general are not. It is noted in passing that the nuclear wavefunction figuring in Equation (19), 〈$\vec{R}$, ϕ^*d*^_*n*_; $\vec{R}$|Ψ〉, differs from that in Equation (11), even through the diabatic transformation operates solely on the electronic states of the system. This is because nuclear wavefunctions result of the projection of the total state of the system onto a particular electronic basis function.

In order to perform the transformation Equation (17), the form of 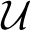 has to be obtained for the desired region of the nuclear coordinate space. This can in principle be done solving Equation (18), which by applying 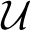 from the left and introducing the identity can be rewritten as

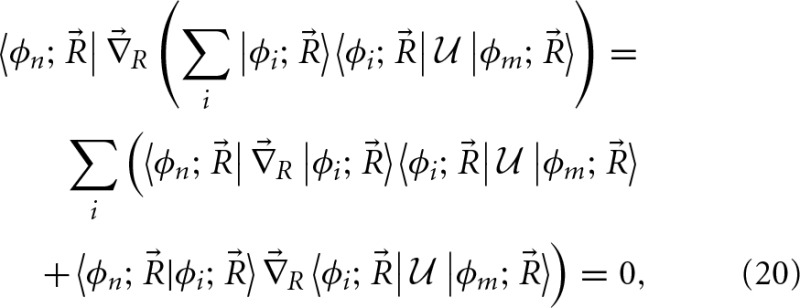



which can be written as the set of differential equations involving derivative couplings and matrix elements of 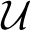

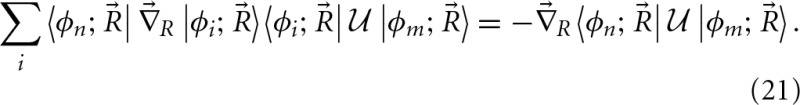




In this equation, the right hand side equals a gradient field, so that in order for the equation to be solvable its curl with respect to the nuclear coordinates should vanish (Arfken and Weber, 2005)

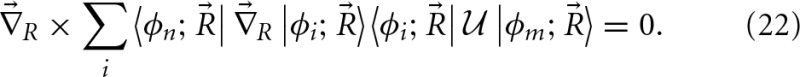



Unfortunately, the condition Equation (22) is in general not fulfilled for more than 1 degree of freedom (Mead and Truhlar, 1982), not even in the case where just the two states at the conical intersection are to be transformed. Thus, no set of strictly-diabatic states satisfying Equation (22) and depending on the nuclear coordinates exists which reduces non-adiabatic couplings to zero^6^. Nonetheless, there are several methods (Delos and Thorson, 1979; Domcke et al., 2004; Baer, 2006) which provide bases which satisfy Equation (18) approximately and in particular have the property that the derivative coupling is no longer singular at conical intersections. Throughout the literature, the “diabatic” designation if often employed to refer to any such quasi-diabatic basis, and this practice will be followed henceforth in this work.

In approximate applications, it is often desirable to build a diabatic representation chosen on physical grounds. Such basis should have a minimum variation with the nuclear coordinates, in order to at least approximately satisfy Equation (18). Appealing candidates here are basis sets based on Valence Bond theory, for which each wavefunction is centered around a particular nucleus (Delos and Thorson, 1979; Garrett and Truhlar, 1981). Motion of the nucleus to which the basis state is associated amounts to a simple translation with conservation of the shape of the wavefunction, and the basis state will be independent of the motion of any other nuclei of the molecule. Valence Bond states have been verified (Sevin et al., 1991) to provide small non-adiabatic coupling values. Additionally, the concept of Valence Bond type states is also particularly useful for the discussion of conical intersection problems in solution involving charge transfer, since it allows the electronic structure to vary with the state of the environment in a simple way (Burghardt et al., 2004; Burghardt and Hynes, 2006; Malhado et al., 2013).

## 4. Conical intersections

Conical intersections (Frey and Davidson, 1990; Yarkony, 2001; Domcke et al., 2004, 2011) are defined as molecular geometries (nuclear configurations) for which the eigenstates of the electronic Hamiltonian Equation (4) are degenerate. Accordingly, as discussed in the preceding section, the non-adiabatic coupling dependence on the electronic energy separation Equation (16) will render the conventional description of the dynamics of the system provided by the Born-Oppenheimer approximation invalid. Non-adiabatic effects will necessarily be present, the dynamics of the different electronic states will be coupled and electronic transitions will be likely to occur at nuclear configurations in the vicinity of the conical intersection.

The shape of the electronic energy surfaces in the vicinity of the intersection—and the origin of the terminology “conical intersection”—are determined by the *non-crossing rule* (Von Neumann and Wigner, 1929; Longuet-Higgins, 1975; Mead, 1979; Hettema, 2000). In general, more than two electronic states can be degenerate at a given nuclear configuration (Coe et al., 2008; Matsika and Krause, 2011), but the focus here will be on the most common case when only two electronic states are involved. In the case of a real electronic Hamiltonian in a real spatial wavefunction representation (and this will be the only case addressed), the non-crossing rule states that the number of degrees of freedom, understood in this context as independent nuclear coordinates, that must be varied in order to induce the degeneracy between two electronic eigenstates of the same symmetry is two (Von Neumann and Wigner, 1929; Longuet-Higgins, 1975; Mead, 1979; Hettema, 2000). This can be seen by considering the matrix representation of such an electronic Hamiltonian in a given basis that spans the intersecting states

(23) $$\left[\begin{array}{cc} H_{11}(\vec{R}) & \;H_{12}(\vec{R})\\ H_{12}(\vec{R}) & \;H_{22}(\vec{R}) \end{array}\right].$$

Most of the results presented in this section are valid for an arbitrary choice of basis set in which to represent the electronic Hamiltonian matrix, but for convenience, it will be assumed that the basis is diabatic. The eigenvalues of Equation (23) represent the electronic energy surfaces

(24) $$\begin{array}{l} V_{\pm }(\vec{R})=\frac{H_{11}(\vec{R})+H_{22}(\vec{R})}{2}\\ \;\pm \;\frac{1}{2}\sqrt{{\left(H_{11}(\vec{R})-H_{22}(\vec{R})\right)}^{2}+4H_{12}{\left(\vec{R}\right)}^{2}}, \end{array}$$

and degeneracy of the two eigenstates will be achieved for *V*_+_($\vec{R}$) = *V*_−_($\vec{R}$), which requires the radical term on the previous equation to be zero, which in turn requires two conditions to be fulfilled:

(25) $$H_{11}(\vec{R})=H_{22}(\vec{R}),$$

(26) $$H_{12}(\vec{R})=0.$$

Since the terms of the matrix Equation (23) are independent, in order to fulfill both conditions simultaneously so that an intersection between the two electronic energy surfaces occurs, it is required that two independent components of $\vec{R}$ are varied. These two components of $\vec{R}$ define a two dimensional subspace of the nuclear coordinates, called the branching space (Atchity et al., 1991), in which the conical intersection is a single point $\vec{R}$_*X*_. It is most convenient (Atchity et al., 1991) to represent this subspace in terms of two coordinates (*u*, *v*) that measure the displacement from the two conditions Equations (25) and (26) that are to be met at $\vec{R}$_*X*_, which are defined along the directions:

(27) $$\vec{u}=\frac{{{\vec{\nabla }}_{R}(H_{11}(\vec{R})-H_{22}(\vec{R}))|}_{{\vec{R}}_{X}}}{\left|{{\vec{\nabla }}_{R}(H_{11}(\vec{R})-H_{22}(\vec{R}))|}_{{\vec{R}}_{X}}\right|};$$

(28) $$\vec{v}=\frac{{{\vec{\nabla }}_{R}H_{12}(\vec{R})|}_{{\vec{R}}_{X}}}{\left|{{\vec{\nabla }}_{R}H_{12}(\vec{R})|}_{{\vec{R}}_{X}}\right|}.$$

The space of nuclear coordinates orthogonal to $\vec{u}$ and $\vec{v}$ has dimension *N* − 2 (where *N* is the number of internal coordinates of the molecular system), is defined by the set of coordinates {*w*} and is called the *intersection space* (Atchity et al., 1991).

In order to usefully exploit these considerations, the electronic Hamiltonian matrix Equation (23) can first be rewritten as the sum

(29) $$\left[\begin{array}{cc} \frac{1}{2}(H_{11}+H_{22}) & 0\\ 0 & \frac{1}{2}(H_{11}+H_{22}) \end{array}\right]+\left[\begin{array}{cc} \frac{1}{2}(H_{11}-H_{22}) & H_{12}\\ H_{12} & -\frac{1}{2}(H_{11}-H_{22}) \end{array}\right]$$

where the explicit dependence on $\vec{R}$ has been suppressed for convenience. For small displacements away from the point $\vec{R}$_*X*_, the elements of these matrices in general may be taken to have a first order dependence on the nuclear coordinates^7^. In this case the electronic Hamiltonian reduces to

(30) $$\left[\begin{array}{cc} A_{u}u+A_{v}v+f(\{w\}) & 0\\ 0 & A_{u}u+A_{v}v+f(\{w\}) \end{array}\right]+\left[\begin{array}{cc} u & v\\ v & -u \end{array}\right]$$

where *A*_*u*, *v*_ = ∂_*u*, *v*_(*H*_11_ + *H*_22_)/2 and *f*({*w*}) is a given function of the intersection space coordinates. The shape of the electronic potential energy surfaces in the vicinity of $\vec{R}$_*X*_ as a function of the defined coordinates *u* and *v* can be obtained from Equation (24) and reads

(31) $$V_{\pm }(u,v,\{w\})=f(\{w\})+A_{u}u+A_{v}v\pm \sqrt{u^{2}+v^{2}}.$$

**Figure 1:**
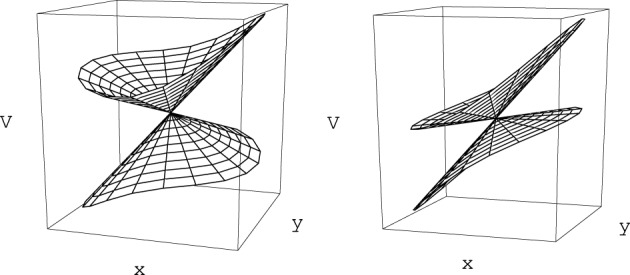
**Electronic energy surfaces form in the vicinity of two generic conical intersections as given by Equation (32) [or equivalently by Equation (33)]**. On the left panel the tilt angle α_*x*_ in Equation (33) is equal to 10° and the conical intersection corresponds to a local minimum of the upper state electronic energy surface (peaked intersection, Atchity et al., 1991). On the right panel α_*x*_ = 50°, the conical intersection in no longer a local energy minimum (sloped intersection, Atchity et al., 1991).

Small displacements along coordinates {*w*} orthogonal to the branching space do not change the gap between the electronic energy surfaces (Sicilia et al., 2007) and thus conserve the degeneracy. In contrast, motion on the branching space coordinates lifts the degeneracy of the electronic eigenstates, and does so linearly with respect to the distance to the intersection point in the neighborhood of that point. When represented with respect to the branching plane (*u*, *v*) coordinates, the surfaces *V*_+_ and *V*_−_ each have a conical shape, with a common apex as displayed in Figure 1. It is this feature that provides the reason why this type of degeneracy is called a conical intersection. While in the branching plane conical intersections are represented by a single point, in the space of the *N* nuclear coordinates these constitute hypersurfaces of dimension *N* − 2, which are termed conical intersection *seams*.

In general, the branching space vectors $\vec{u}$ and $\vec{v}$ defined in Equations (27) and (28) are not orthogonal and thus are not the most convenient to study the dynamical properties of the system. This is easily remedied by orthogonalization, rotation, and scaling by appropriate mass factors (Atchity et al., 1991) such that in the resulting orthogonal coordinates (*x*, *y*), which also define the branching space, Equation (31) assumes the form

(32) $$V_{\pm }(x,y)=A_{x}x+A_{y}y\pm \sqrt{B_{x}x^{2}+B_{y}y^{2}}$$

where the dependence on the remaining {*w*} coordinates has been suppressed. In a very convenient representation of the branching plane in polar coordinates $r=\sqrt{x^{2}+y^{2}}$ and θ = arctan(*y*/*x*), the previous equation can be written as

(33) $$\begin{array}{l} V_{\pm }(r,\theta )=Fr(\tan(\alpha_{x})\cos(\theta )+\tan(\alpha_{y})\sin(\theta )\\ \;\pm \;\sqrt{\cos^{2}(\theta )+e\sin^{2}(\theta )}), \end{array}$$

with a matrix representation of the electronic Hamiltonian given by

(34) $$\begin{array}{l} Fr(\left(\tan(\alpha_{x})\cos(\theta )+\tan(\alpha_{y})\sin(\theta )\right)𝕀\;\;\\ \;+\;\left[\begin{array}{cc} \cos(\theta ) & \sqrt{e}\sin(\theta )\\ \sqrt{e}\sin(\theta ) & -\cos(\theta ) \end{array}\right]), \end{array}$$

where 𝕀 is the identity matrix. Equation (33) represents a general double cone surface where *r* represents the distance to the apex, *F* defines a general slope along *r*, the coefficients have been written as a function of tilt angles α_*x*_ and α_*y*_, and *e* represents the elliptical deformation of the cone. For values of the tilt angles α_*x*_ and α_*y*_ smaller than π/4 the conical intersection point corresponds to a local minimum of the excited state surface. This is an important case since, as will be made clearer in the following sections, the excited state potential energy surface drives the system toward a conical intersection nuclear configuration.

A notable property of the surfaces described by Equation (33) is that the upper and lower surfaces represent the continuation of each other in the sense that for any given value θ

(35) $${\frac{\partial V_{+}(r,\theta )}{\partial r}|}_{r\;=\;0}=-{\frac{\partial V_{-}(r,\theta +\pi )}{\partial r}|}_{r\;=\;0}$$

(see Figure 1). This property is also related to the geometric phase effect (Longuet-Higgins, 1961; Herzberg and Longuet-Higgins, 1963; Berry, 1984; Bohm, 1993) which is characteristic of conical intersections, not present in avoided crossings or intersections of a different type (see footnote 7). Geometric phase will be briefly discussed in Section 4.1.

While the non-crossing rule determines that conical intersections are manifolds of measure zero on the nuclear coordinate space, they are limited to one point in the branching space, and at this point the non-adiabatic derivative coupling vectors are singular according to Equation (16), the electronic energy gap will remain small in a finite region of the nuclear coordinate space around the conical intersection, and so will the magnitude of these vectors. It is the configuration and magnitude of the derivative non-adiabatic coupling vectors in the vicinity of conical intersections which are responsible for these features' influence on the dynamics of molecular systems through Equation (11). These will be analyzed in more detail in Section 4.2.

### 4.1. Manifestation of the geometric phase

A simple and important illustration of the geometric phase effect can be seen by representing the basis set unitary transformation that diagonalizes the electronic Hamiltonian matrix Equation (23) as a rotation in the space of the electronic states to give the eigenvalues Equation (24)

(36) $$\left[\begin{array}{cc} \cos(\lambda ) & -\sin(\lambda )\\ \sin(\lambda ) & \cos(\lambda ) \end{array}\right]\left[\begin{array}{cc} H_{11} & H_{12}\\ H_{12} & H_{22} \end{array}\right]\left[\begin{array}{cc} \cos(\lambda ) & \sin(\lambda )\\ -\sin(\lambda ) & \cos(\lambda ) \end{array}\right]=\left[\begin{array}{cc} V_{+} & 0\\ 0 & V_{-} \end{array}\right].$$

By equating any of the terms of the matrix equation [and using Equation (24) in the case of diagonal terms] an expression for the rotation parameter is obtained

(37) $$\tan(2\lambda )=\frac{2H_{12}}{H_{11}-H_{22}}.$$

When the nuclear position dependence of the electronic Hamiltonian matrix in the vicinity of a conical intersection Equation (34) is inserted in the last equation, a relation to the nuclear geometry is obtained in the form

(38) $$\lambda (\theta )=\frac{1}{2}\arctan\left(\sqrt{e}\tan(\theta )\right).$$

Although it has the attractive feature of being very compact, this equation is problematic due to the discontinuities of the tan function. Using the identity involving the arctan function (Abramowitz and Stegun, 1972)

(39) $$\arctan(A)-\arctan(B)=\arctan\left(\frac{A-B}{1+AB}\right),$$

it is possible to rewrite Equation (38) in the more convenient form

(40) $$\lambda (\theta )=\frac{1}{2}\left(\arctan\left(\frac{\left(\sqrt{e}-1)\sin(\theta )\cos(\theta \right)}{\cos^{2}(\theta )+\sqrt{e}\sin^{2}(\theta )}\right)+\theta \right).$$

Equation (40) relates the “angle” λ which determines the linear combination of basis states that diagonalize the electronic Hamiltonian, with the angle θ that defines a rotation in the branching space of nuclear configurations around the conical intersection. The equation reveals one aspect of what is termed the geometric phase effect, since it shows that a full rotation in the nuclear space around the conical intersection, θ = 2π, implies a change in sign of the electronic eigenstate, λ = π. This can be seen by considering, for example, the lower adiabatic state written in a two state diabatic basis, and explicitly singling out the nuclear position dependence on the θ angle, this eigenstate sign reversal reads

(41) $$\begin{array}{l} |\phi_{-};\theta +2\pi \rangle =\cos(\lambda +\pi )|\phi_{1}^{d};\theta +2\pi \rangle \\ \;+\;\sin(\lambda +\pi )|\phi_{2}^{d};\theta +2\pi \rangle \\ \;=-\left(\cos(\lambda )|\phi_{1}^{d};\theta \rangle +\sin(\lambda )|\phi_{2}^{d};\theta \rangle \right)\\ \;=-|\phi_{-};\theta \rangle . \end{array}$$

Since when keeping all other coordinates fixed, the nuclear position θ is obviously the same as θ + 2π, this equation shows that the electronic eigenfunctions are not single-valued. This fact needs to be taken into account in the quantum mechanical description of the nuclear dynamics (Longuet-Higgins, 1961; Yarkony, 2001; Juanes-Marcos et al., 2005) when using an electronic eigenstate representation such as the Born-Oppenheimer expansion Equation (7). However, the focus of the present work will be on a classical description of the nuclear motion, as will be further detailed in Section 5 and the following sections, and issues related to the geometric phase will not play a role.

Nonetheless, it is noted that the geometric phase affect has a useful practical application. Although Equation (41) was derived invoking an electronic Hamiltonian matrix of the form Equation (34), which is valid in the vicinity of a generic conical intersection, the result is more general and is a consequence of the topological characteristics of the electronic energy surfaces (Longuet-Higgins, 1975). The inversion of the sign of the electronic adiabatic wavefunction upon completion of a loop in nuclear coordinate space encircling the conical intersection is a characteristic signature of its presence (Herzberg and Longuet-Higgins, 1963; Frey and Davidson, 1990), and can be observed in electronic structure calculations (Varandas et al., 1979; Ceotto and Gianturco, 2000; Vanni et al., 2008).

### 4.2. Non-adiabatic coupling in the vicinity of a conical intersection

As discussed in Section 2, the non-adiabatic dynamics of a molecular system and the coupling between electronic adiabatic states is due to the non-adiabatic coupling terms, and in particular the derivative non-adiabatic coupling vectors. It is thus useful to look in detail at the shape of the vectors field formed by these quantities in the vicinity of conical intersection. In order to do this, it is convenient to proceed by first projecting the electronic states into a diabatic basis |ϕ^*d*^_*i*_; $\vec{R}$〉

(42) $$\begin{array}{l} \langle \phi_{n};\vec{R}|{\vec{\nabla }}_{R}|\phi_{m};\;\vec{R}\rangle \;\\ =\sum_{ij}\langle \phi_{n};\vec{R}|\phi_{i}^{d};\vec{R}\rangle \langle \phi_{i}^{d};\vec{R}|{\vec{\nabla }}_{R}\left(|\phi_{j}^{d};\vec{R}|\phi_{m};\vec{R}\rangle \right)\;\\ =\sum_{ij}(\langle \phi_{n};\vec{R}|\phi_{i}^{d};\vec{R}\rangle \langle \phi_{i}^{d};\vec{R}|{\vec{\nabla }}_{R}|\phi_{j}^{d};\vec{R}\rangle \langle \phi_{j}^{d};\vec{R}|\phi_{m};\vec{R}\rangle \\ \;+\langle \phi_{n};\vec{R}|\phi_{i}^{d};\vec{R}\rangle \langle \phi_{i}^{d};\vec{R}|\phi_{j}^{d};\vec{R}\rangle {\vec{\nabla }}_{R}\langle \phi_{j}^{d};\vec{R}|\phi_{m};\vec{R}\rangle ). \end{array}$$

An equation for determining the non-adiabatic coupling terms from the coefficients of the expansion of the adiabatic states in the diabatic basis is obtained by noting that the first term inside the sum is zero by the definition of diabatic states [see Equation (18)], and then observing that since the diabatic states also form an orthonormal basis, the second term reduces to a single summation

(43) $$\langle \phi_{n};\vec{R}|{\vec{\nabla }}_{R}|\phi_{m};\;\vec{R}\rangle =\sum_{i}\langle \phi_{n};\vec{R}|\phi_{i}^{d};\vec{R}\rangle {\vec{\nabla }}_{R}\langle \phi_{i}^{d};\vec{R}|\phi_{m};\vec{R}\rangle .$$

Each one of the bra-ket terms in this equation corresponds to the matrix elements of the unitary adiabatic-to-diabatic basis transformation defined in Equation (17), 〈ϕ_*n*_; $\vec{R}$|
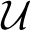
|ϕ_*m*_; $\vec{R}$ = 〈ϕ_*n*_; $\vec{R}$|ϕ^*d*^_*m*_; $\vec{R}$〉. In the two state case, with the unitary transformation 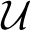 represented as a rotation matrix as in Equation (36) and the gradient expressed in polar coordinates, the derivative non-adiabatic coupling 〈ϕ_+_; $\vec{R}$|$\vec{\nabla }$_*R*_|ϕ_−_; $\vec{R}$〉 = −〈ϕ_−_; $\vec{R}$|$\vec{\nabla }$_*R*_|ϕ_+_; $\vec{R}$〉 can be written as

(44) $$\begin{array}{l} \langle \phi_{+};\vec{R}|{\vec{\nabla }}_{R}|\phi_{-};\;\vec{R}\rangle =\\ \;(\cos(\lambda )\left(\vec{e_{r}}\frac{\partial }{\partial r}(-\sin(\lambda ))+\vec{e_{\theta }}\frac{1}{r}\frac{\partial }{\partial \theta }(-\sin(\lambda ))\right)\\ \;+\sin(\lambda )\left(\vec{e_{r}}\frac{\partial }{\partial r}\cos(\lambda )+\vec{e_{\theta }}\frac{1}{r}\frac{\partial }{\partial \theta }\cos(\lambda )\right)), \end{array}$$

where $\vec{e}$_*r*_ and $\vec{e}$_θ_ are unit vectors along the radial and angular directions, respectively. Since by Equation (40) the parameter λ does not depend on the radial coordinate *r*, the previous equation considerably simplifies to

(45) $$\langle \phi_{+};\vec{R}|{\vec{\nabla }}_{R}|\phi_{-};\;\vec{R}\rangle =-\frac{1}{r}\frac{\partial \lambda }{\partial \theta }\vec{e_{\theta }}.$$

**Figure 2:**
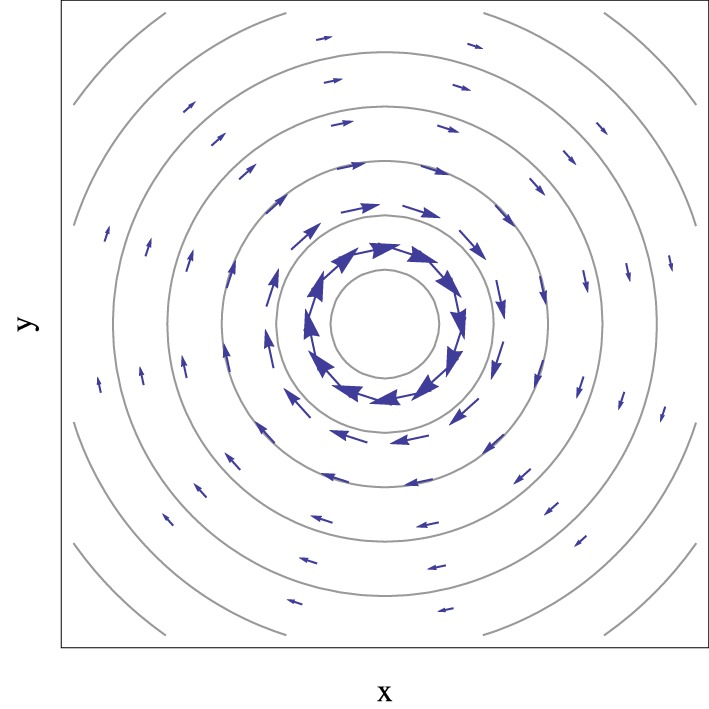
**Representation of the derivative non-adiabatic coupling vector field, given by Equation (45), in the vicinity of a conical intersection described by Equation (33), with the apex at the center and α_*x*_ = α_*y*_ = 0 and *e* = 1**. Contour lines represent the electronic eigenstate energy gap and the arrow size is proportional to the magnitude of non-adiabatic coupling vectors.

Equation (45) shows that the derivative non-adiabatic coupling field, represented in Figure 2, has only a tangential component in the branching plane in the vicinity of conical intersections, and its magnitude decreases with the inverse of the distance to the degeneracy point [as is implied by the inverse dependence on the electronic eigenstate energy gap Equation (16) together with the representation Equation (33)]. Because of this dependence it is also possible, and often convenient, to define the branching space as a function of the derivative non-adiabatic coupling vector [or only the numerator in Equation (16) which sets its direction] and the adiabatic energy gap gradient (Yarkony, 1996, 1998, 2001) defined at a given point in the close vicinity of the conical intersection instead of the vectors in Equation (27). In this case, the common notation is $\vec{h}$ and $\vec{g}$ for each coordinate, respectively.

To the extent that non-adiabatic effects at conical intersections are associated with the derivative non-adiabatic coupling, Figure 2 shows that this effect is not limited to the strict points of degeneracy but rather the volume (an area in the branching space) of nuclear space surrounding it, and that it will depend on the characteristic direction of the vector field. In the next sections it will be shown that this fact has important implications for the non-adiabatic dynamics of the system, in particular when the motion of the nuclei is described classically.

## 5. Electronic dynamics with classical nuclei

The description given in the previous sections is based on a quantum mechanical treatment of both the electrons and nuclei comprising a molecule. The Born-Oppenheimer expansion and approximation, described in Section 2, build upon the differences in masses, and consequent differences in the time scale of the dynamics, of electrons and nuclei. A further approximation in this vein is to consider that the dynamics of the nuclei can be described by classical mechanics; in this case the system will be defined at each instant by a point in the classical phase space consisting of the positions and momenta of the nuclei and a quantum state determined by the quantum electronic Hamiltonian Equation (4) ^8^.

In general, and in the particular case of conical intersections, the dynamics of the classical part of such a quantum-classical interacting system will depend on a classical Hamiltonian, whose definition is however not straightforward regarding the quantum system's influence on the classical motion. The discussion of the determination of the nuclear classical trajectories is delayed to Section 7 and for the purposes of the discussion in the present section is assumed to be the generic function of time $\vec{R}$(*t*).

The evolution of the system's quantum part is given by the Schrödinger equation involving the electronic Hamiltonian Equation (4), which in this context becomes dependent on time via the nuclear coordinates $\vec{R}$(*t*) becoming a time dependent parameter

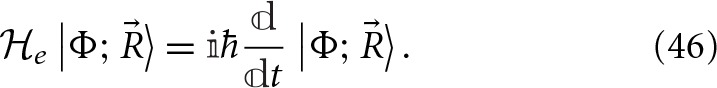



Solutions of this equation may be expanded in terms of an arbitrary orthonormal basis |φ_*i*_; $\vec{R}$〉

(47) $$|\Phi ;\;\vec{R}\rangle =\sum_{m}|\varphi_{m};\vec{R}\rangle \langle \varphi_{m};\vec{R}|\Phi ;\vec{R}\rangle .$$

Using this expansion in Equation (46) and taking the scalar product with an element of the basis, one obtains

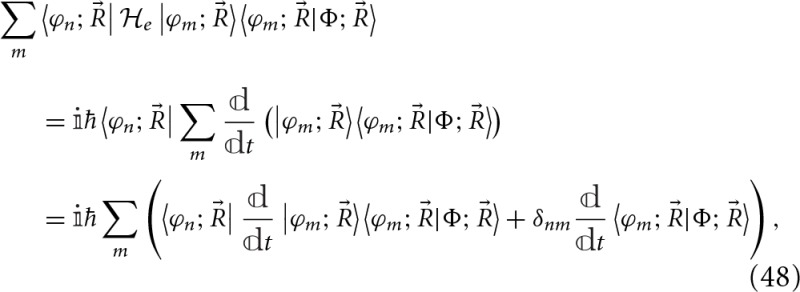



which constitutes a set of differential equations for the coefficients 〈φ_*n*_; $\vec{R}$|Φ; $\vec{R}$〉 (Nikitin, 1974). After rearrangement to isolate the time derivative of the coefficient, this yields

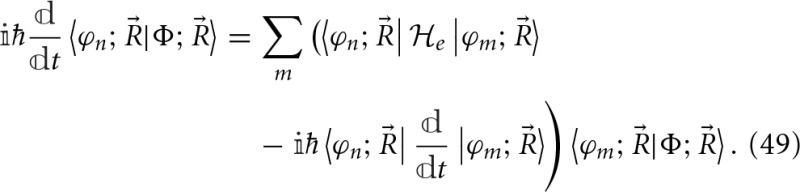



Since the electronic Hamiltonian depends on time through the nuclear coordinates as do the basis elements |φ_*i*_; $\vec{R}$〉, the second coupling term in Equation (49) can be expressed in terms of the derivative non-adiabatic coupling, discussed in Section 2 and the nuclear velocity:

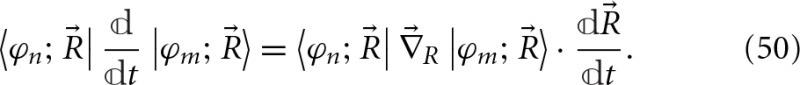



A pause is made here in order to highlight a relevant difference with respect to the full quantum treatment followed in Sections 2 and 3 and the current quantum-classical one. In the former, the coefficients 〈$\vec{R}$, ϕ_*n*_; $\vec{R}$|Ψ〉 in Equation (11) and 〈$\vec{R}$, ϕ^*d*^_*n*_; $\vec{R}$|Ψ〉 Equation (19) are coefficients of a state defined in a Hilbert space which includes the space of the nuclei motion 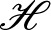
_*slow*_ [Equation (3)] and thus also include information on the motion of the nuclei. For the latter, the coefficients 〈φ_*n*_; $\vec{R}$|Φ; $\vec{R}$〉, while depending on the nuclear coordinates, are associated only with the electron motion 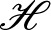
_*fast*_, and all information on the nuclear motion is defined at each instant by a point in the classical phase space. It is due to this difference that states |φ_*n*_; $\vec{R}$〉 are in general time-dependent while states |$\vec{R}$, ϕ_*n*_; $\vec{R}$〉 and |$\vec{R}$, ϕ^*d*^_*n*_; $\vec{R}$〉 are not [see Equation (10)]. (Another important consequence of the reduction of the nuclear degrees of freedom to a phase space description will be further discussed in Section 7.2).

Returning to the equation of motion Equation (49), in order to simplify it proves convenient (Nikitin, 1974) to factor out phase terms of the form 
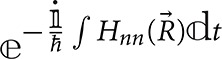
, where for notational convenience *H*_*nn*_($\vec{R}$) = 〈φ_*n*_; $\vec{R}$|
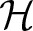
_*e*_|φ_*n*_; $\vec{R}$〉 is explicitly extracted from the coefficients 〈φ_*n*_; $\vec{R}$|Φ; $\vec{R}$〉, such that a new coefficient *c*_*n*_ is defined by

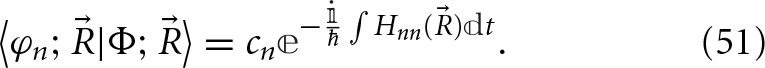



Introduction of definition Equation (51) into Equation (49) then gives

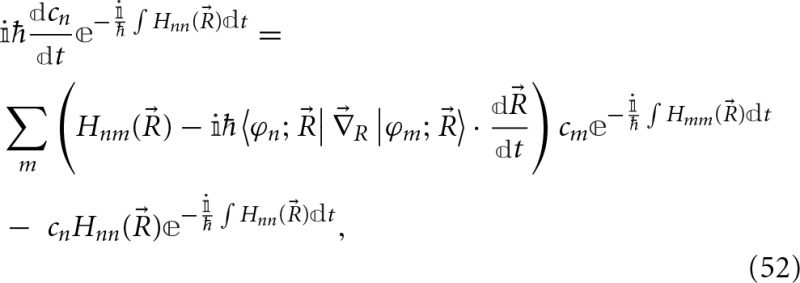



and by noting that the terms 〈φ_*n*_; $\vec{R}$|$\vec{\nabla }$_*R*_|φ_*m*_; $\vec{R}$〉 form a real anti-hermitian matrix which has zeros along the diagonal, one obtains

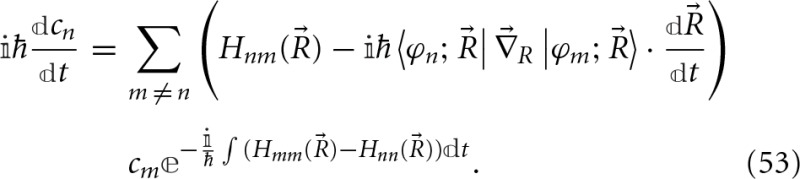



These equations form a set of coupled differential equations for the evolution of the quantum system as a function of the classic trajectory $\vec{R}$(*t*) of the nuclei (Nikitin, 1974).

Two choices of electronic basis |φ_*m*_; $\vec{R}$〉 discussed previously simplify Equation (53); these are the adiabatic basis of eigenstates |ϕ_*m*_; $\vec{R}$〉 of the electronic Hamiltonian first discussed in Section 2, in which its matrix representation is diagonal and the diabatic basis |ϕ^*d*^_*m*_; $\vec{R}$〉, discussed in Section 3, in which non-adiabatic coupling terms 〈φ_*n*_; $\vec{R}$|$\vec{\nabla }$_*R*_|φ_*m*_; $\vec{R}$〉 are zero:

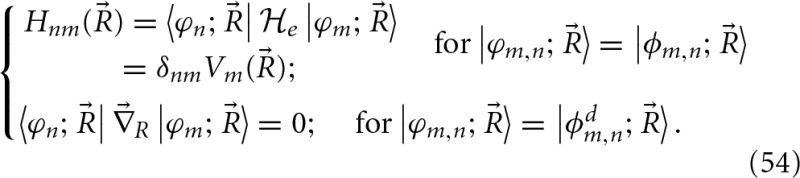



The diabatic representation will be used in Section 6 in the derivation of the Landau-Zener expression. In this representation, Equation (53) becomes

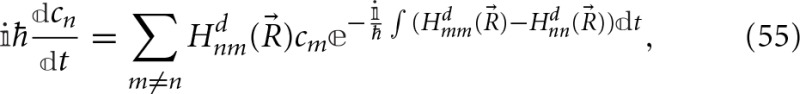



where the coupling between electronic states is due to the off-diagonal elements of the electronic Hamiltonian matrix, as in Equation (19). The adiabatic representation will be used in the surface hopping description of non-adiabatic dynamics in Section 7. Now Equation (53) reduces to

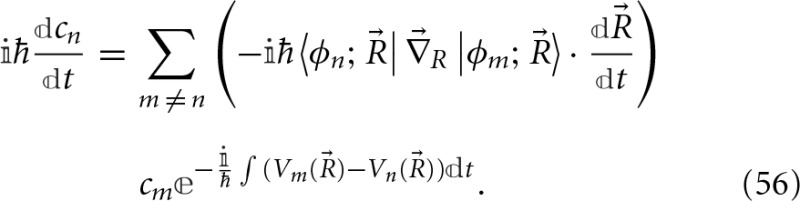



The coupling in Equation (56) is due to derivative non-adiabatic coupling terms, a feature shared with the full quantum Equation (11), although in contrast with Equation (11), no kinetic non-adiabatic coupling terms are present. Due to Equation (16), coefficients of the electronic eigenstates will vary the most at nuclear configurations where differences of the electronic eigenvalues are small, such as in the vicinity of conical intersections. However, the coupling between electronic eigenstates does not simply depend on their energy difference. This is most clear in Equation (56), where the electronic eigenstates are coupled by the nuclear motion through the inner product of the derivative non-adiabatic coupling vector and the nuclear velocity. This quantity has an intrinsic directional character and it will be more significant at high nuclear velocities and when the derivative coupling and the nuclear velocity have the same direction.

**Figure 3:**
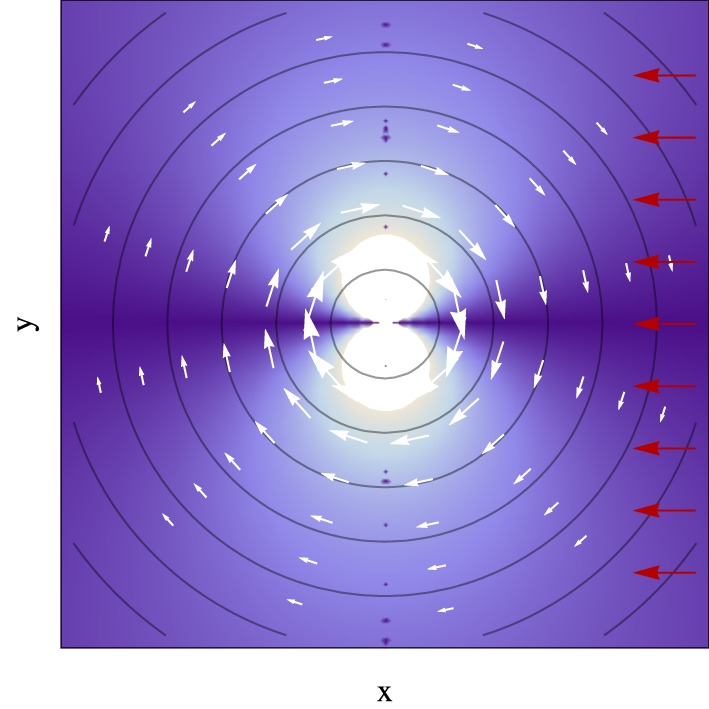
**Absolute value of the inner product of the derivative non-adiabatic coupling vector field (white arrows) in the vicinity of a conical intersection given by Equation (33), with the apex at the center and α_*x*_ = α_*y*_ = 0 and *e* = 1, with a uniform nuclear velocity with no component along the *y* axis (red arrows)**. Brighter color represents higher inner product magnitude. Contour lines represent the electronic eigenstate energy gap, with the conical intersection located at the center of the plot.

In most cases in the context of photochemistry, the system will approach the conical intersection from a given direction in the nuclear coordinate space. Figure 3 shows an illustrative case which assumes a uniform velocity how the coupling between electronic eigenstates varies as a function of space in the vicinity of a conical intersection. It is revealing that while in this case the energy difference increases linearly with the distance to the degeneracy and does not depend on the direction, the coupling strongly does, vanishing along the direction of the velocity pointing to the conical intersection, and with its maximum along the direction perpendicular to the velocity. As will be further discussed in Section 7.1, this directional nature of the coupling has consequences for the nuclear position where non-adiabatic transitions occur.

## 6. Landau-Zener equation

The previous section set out the differential equations that govern the quantum electronic dynamics for a system with a classical nuclear nuclear trajectory. An important question to answer with these equations: in a two state system, what will be the probability of, when measured in one of the electronic eigenstates, to be measured on the other electronic eigenstate after the nuclear trajectory has passed through an area of non-adiabaticity? In other words, what is the probability of non-adiabatic transitions between electronic eigenstates? Answering this question involves solving Equations (55) or (56), which will have close-form solutions only in specific cases. The Landau-Zener (LZ) model (Landau, 1932; Majorana, 1932; Stückelberg, 1932; Zener, 1932) provides an equation for the probability of non-adiabatic transitions. As will be discussed toward the end of the current section, the model is both applicable for a trajectory passing in the close vicinity of a conical intersection (despite the fact that it is often thought not to be), and instructive from the point of view of describing which quantities affect this probability. In the following, the LZ model is described and the equation for the non-adiabatic transition probability is derived.

The LZ model applies to the case where only two electronic eigenstates are close in energy for the region of nuclear space of interest, with all other states well-separated in energy and, from Equations (16) and (11) or (53), decoupled from the two states of interest. Further, areas where the energy surfaces of these states have a small energy gap [whether or not they are in the close vicinity of conical intersections (Truhlar and Mead, 2003)] should constitute limited regions of the nuclear coordinate space. It proves more useful to work with the trajectory's arc-length *z*(*t*) rather than with the classical trajectory $\vec{R}$(*t*) itself. The former can be calculated as the integral of the magnitude of the velocity

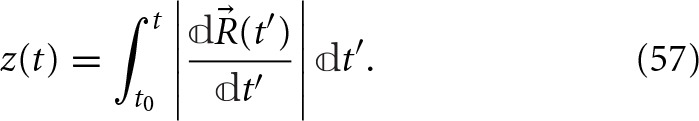



The LZ model considers the case of a two states system which has a diabatic representation, |ϕ^*d*^_*n*_; $\vec{R}$〉, that becomes degenerate at some nuclear configuration along the trajectory *z*_*c*_ (i.e., equal diagonal elements of the electronic Hamiltonian matrix *H*^*d*^ at the point *z*_*c*_). For configurations away from *z*_*c*_, these diabatic states coincide with the adiabatic eigenstates of the electronic Hamiltonian |ϕ_*n*_; $\vec{R}$〉. These conditions can be stated as the requirement that the matrix representation of the electronic Hamiltonian in diabatic representation—which is in general non-diagonal—should tend asymptotically to a diagonal representation:

(58) $$\underset{|\phi_{n}^{d};\;\vec{R}\rangle }{\left[\begin{array}{cc} H_{11}^{d}(z) & \;H_{12}^{d}(z)\\ H_{12}^{d}(z) & \;H_{22}^{d}(z) \end{array}\right]}\overset{}{\underset{|z\;-\;z_{c}|\to \infty }{\to }}\underset{|\phi_{n};\;\vec{R}\rangle }{\left[\begin{array}{cc} V_{+}(z) & 0\\ 0 & V_{-}(z) \end{array}\right]}$$

For any value of *z* the diabatic electronic Hamiltonian matrix can always be diagonalized, with the resulting diagonal elements given by Equation (24). Equations (24) and (58) imply the consistency condition

(59) $$\underset{|z(t)-z_{c}|\to \infty }{\lim}\frac{H_{12}^{d}(z)}{\left|H_{22}^{d}(z)-H_{11}^{d}(z)\right|}=0.$$

Condition (59) [a consequence of (58] should be seen as a requirement that the diabatic basis should fulfill, namely that the regions where *H*^*d*^_12_ is significant should be well-localized along the trajectory.

**Figure 4:**
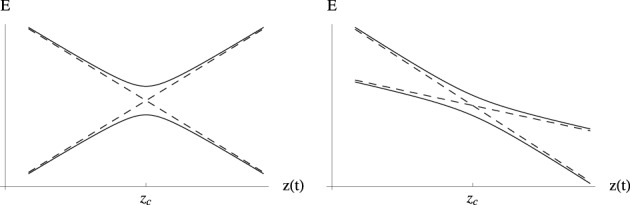
**Energy profiles of the diagonal elements of the diabatic (- -) and adiabatic (–) matrices Equation (58) for the LZ model as a function of the nuclear trajectory length *z*(*t*)**. *z*_*c*_ corresponds to the configuration along the trajectory for which the diabatic states are degenerate and where the energy gap between adiabatic states is minimal. Geometrically the adiabatic curves are hyperbolas and the diabatic curves correspond to their asymptotes. The left hand side panel presents a case where the diabatic state energy variations with *z*(*t*) have slopes of different sign, while on the right hand side both slopes are negative.

In the LZ model, all elements of the diabatic Hamiltonian matrix *H*^*d*^ should have at most a linear variation with the trajectory length, and in particular, the diagonal elements of the matrix *H*^*d*^ are taken to diverge linearly in *z*(*t*) away from *z*_*c*_ (see Figure 4). These conditions translate into first order Taylor series expansions around *z*_*c*_:

(60) $$H_{12}^{d}(z)=H_{12}^{d}(z_{c})+{\frac{\partial H_{12}^{d}(z)}{\partial z}|}_{z\;=\;z_{c}}(z-z_{c});$$

(61) $$\begin{array}{l} H_{22}^{d}(z)-H_{11}^{d}(z)=(H_{22}^{d}(z_{c})-H_{11}^{d}(z_{c}))\\ \;+{\frac{\partial \left(H_{22}^{d}(z)-H_{11}^{d}(z)\right)}{\partial z}|}_{z\;=\;z_{c}}(z-z_{c}). \end{array}$$

However, the first term on the right hand side of Equation (61) is zero by definition of the point *z*_*c*_. Further, given the dependence of *z*(*t*) of both Equations (60) and (61), condition Equation (59) can only hold if the slope of the off-diagonal terms vanishes

(62) $${\frac{\partial H_{12}^{d}(z)}{\partial z}|}_{z\;=\;z_{c}}=0,$$

so that the electronic coupling between diabatic states must be taken to be constant. Thus, the dependence of diabatic matrix elements on the nuclear trajectory length can be written as

(63) $$H_{12}^{d}(z)=H_{12}^{d}(z_{c})=H_{12}^{d};$$

(64) $$\begin{array}{l} \;H_{22}^{d}(z)-H_{11}^{d}(z)={\frac{\partial \left(H_{22}^{d}(z)-H_{11}^{d}(z)\right)}{\partial z}|}_{z\;=\;z_{c}}(z-z_{c})\\ \;=\Delta S(z-z_{c}), \end{array}$$

where Δ*S* can be seen as the difference in slopes, *S*_1_ and *S*_2_, of each diagonal element of the matrix as a function of *z*(*t*). In the LZ model then, the diagonalization of the electronic Hamiltonian through Equation (24) gives a hyperbolic dependence of the eigenvalues *V*_±_, i.e., the adiabatic state energy profile, as a function of *z*(*t*), where *H*^*d*^_11_($\vec{R}$) and *H*^*d*^_22_($\vec{R}$) will be the asymptotes of the hyperbolas (Figure 4)

(65) $$\begin{array}{l} V_{\pm }(z)=\frac{1}{2}(H_{11}^{d}(z_{c})+H_{22}^{d}(z_{c})+(S_{1}+S_{2})(z-z_{c}))\\ \;\pm \frac{1}{2}\sqrt{\Delta S{\left(z-z_{c}\right)}^{2}+4{\left(H_{12}^{d}\right)}^{2}}. \end{array}$$

The minimum adiabatic energy gap Δ*V* along *z*(*t*) will occur at *z*_*c*_ with its value equal to twice the magnitude of the electronic coupling between the diabatic states:

(66) $$\Delta V=|V_{+}(z_{c})-V_{-}(z_{c})|=2|H_{12}^{d}|.$$

The probability to measure the system |Φ; $\vec{R}$〉 in higher energy electronic eigenstate |ϕ_+_; $\vec{R}$〉 for a value $\vec{R}$(*t*_0_) = $\vec{R}$_0_ in a point along the trajectory preceding *z*_*c*_ is the squared amplitude the eigenstate expansion coefficient |〈ϕ_+_; $\vec{R}$_0_|Φ; $\vec{R}$_0_|^2^. Similarly, the probability of measuring the system in lower energy eigenstate |ϕ_−_; $\vec{R}$〉 for a nuclear configuration $\vec{R}$(*t*_∞_) = $\vec{R}$_∞_ having passed beyond the point *z*_*c*_, is |〈ϕ_−_; $\vec{R}$_∞_|Φ; $\vec{R}$_∞_|^2^. The probability *p*_*hop*_ of a non-adiabatic electronic transition after crossing a region of strong coupling, a “hop,” is then

(67) $$p_{hop}={\left|\langle \phi_{-};{\vec{R}}_{\infty }|\Phi ;{\vec{R}}_{\infty }\rangle \right|}^{2};\text{ for}{\left|\langle \phi_{+};{\vec{R}}_{0}|\Phi ;{\vec{R}}_{0}\rangle \right|}^{2}=1.$$

For the LZ model, given the properties of the diabatic basis defined by Equation (58), |ϕ^*d*^_1_; $\vec{R}$_0_〉 ≈ |ϕ_+_; $\vec{R}$_0_〉 and |ϕ^*d*^_1_; $\vec{R}$_∞_〉 ≈ |ϕ_−_; $\vec{R}$_∞_〉, the probability of a non-adiabatic electronic transition between adiabatic states is

(68) $$p_{hop}={\left|\langle \phi_{1}^{d};{\vec{R}}_{\infty }|\Phi ;{\vec{R}}_{\infty }\rangle \right|}^{2};\text{ for}{\left|\langle \phi_{1}^{d};{\vec{R}}_{0}|\Phi ;{\vec{R}}_{0}\rangle \right|}^{2}=1.$$

Determining *p*_*hop*_ thus involves the determination of the evolution of the coefficient of the electronic state 〈ϕ^*d*^_1_; $\vec{R}$|Φ; $\vec{R}$〉 in the diabatic basis, or equivalently *c*_1_ defined in Equation (51), from *t* = *t*_0_ to *t* = *t*_∞_ with the initial condition of certain occupation *c*_1_(*t*_0_) = 1. This can be done using the evolution Equation (55), which for a two state model in the diabatic representation can be written as

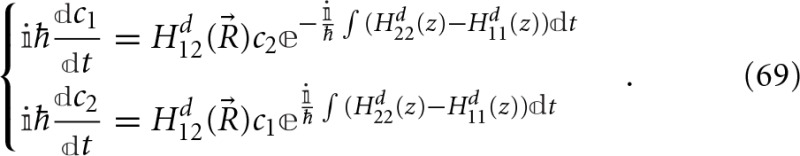



Making use of the LZ model's variation of the elements of Hamiltonian in the diabatic basis with *z*(*t*) Equations (63) and (64), and introducing the further approximation that the magnitude of the velocity along the trajectory is constant, 
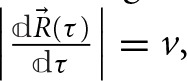
, Equation (57) becomes ^9^

(70) $$(z(t)-z_{c})=vt,$$

and the evolution Equation (69) can be rewritten as

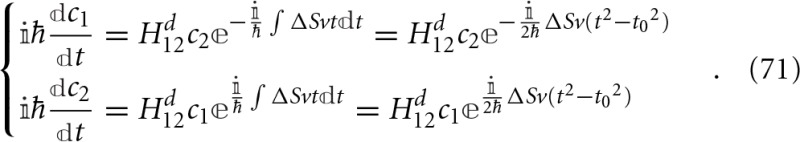



Finding the electronic transition probability *p*_*hop*_ consists in solving this system of differential equations. Landau (1932), (Zener, 1932), (Stückelberg, 1932), and (Majorana, 1932) each follow a different approach to the solution of the problem^10^. Briefly sketched, Zener's solution (Zener, 1932), which is perhaps more explicit in reference (Heinrichs, 1968), involves transforming the system of two coupled first order Equation (71) into a single second order equation by solving the first equation of the set in order to determine *c*_2_ as a function of the *c*_1_ time derivative and substituting this into the second equation. This procedure yields

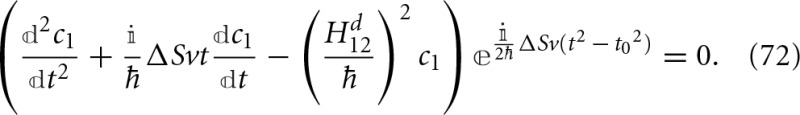



By making the substitution 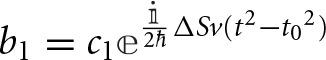, and a variable substitution from *t* to *x* by rotating the time axis onto the complex plane 
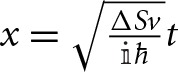
, the previous equation can be written as a Weber parabolic cylinder equation (Whittaker and Watson, 1927; Abramowitz and Stegun, 1972)

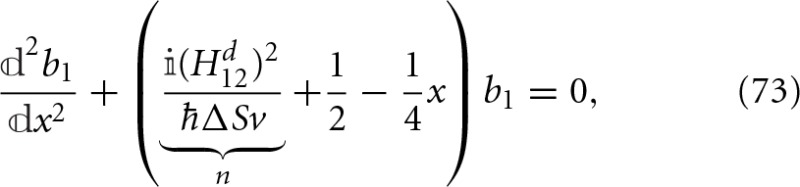



where *n* is a pure imaginary constant. An expression for the transition probability *p*_*hop*_ can be obtained by manipulating asymptotic expansions of Weber parabolic cylinder functions, *D*_*n*_(*x*), in the limit of long times (Zener, 1932; Heinrichs, 1968), the final result being

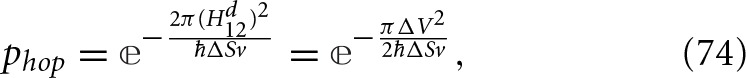



where in the second equality Equation (66) connecting the diabatic coupling with the adiabatic energy gap was used.

The key predictions of the LZ Equation (74) are the following. The probability *p*_*hop*_ of an electronic transition between adiabatic states, along a given nuclear path, increases exponentially as the square of the minimum energy gap Δ*V* between these electronic adiabatic states decreases. This probability also increases exponentially as the slope difference Δ*S* of the diabatic states energy profiles increases. Finally, *p*_*hop*_ increases exponentially with increasing velocity with which the non-adiabatic region is crossed. (Here it is useful to recall from Equation (53) that the coupling between adiabatic states is proportional to the velocity.)

**Figure 5:**
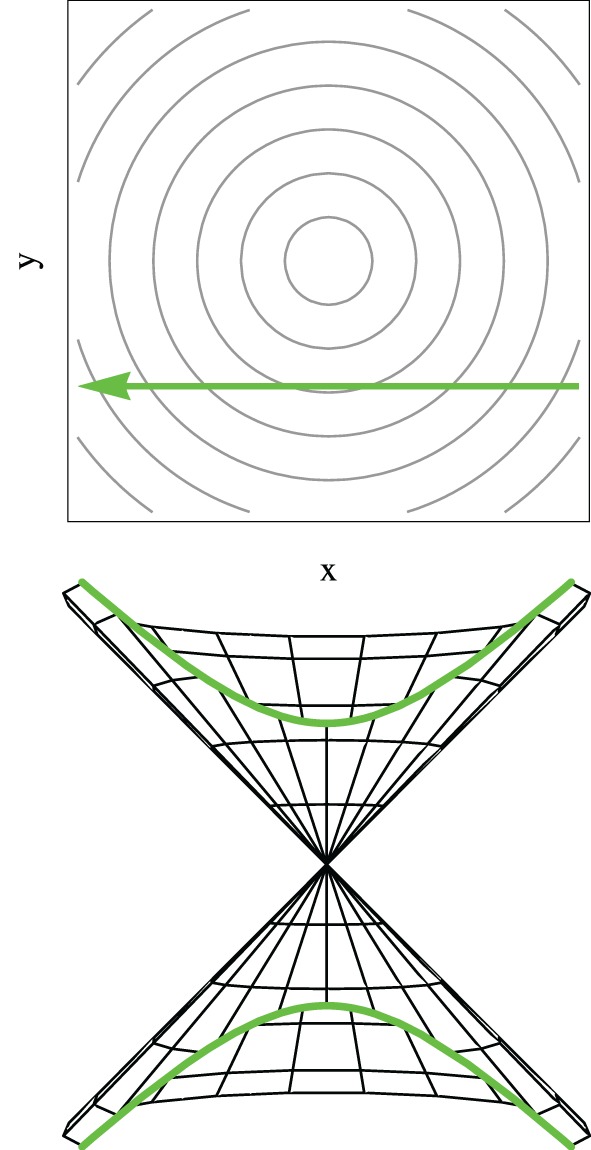
**The adiabatic potential energy profile for a straight line trajectory on the branching space for potential energy surfaces given by Equation (32) [or equivalently by Equation (33)] is hyperbolic**. The upper panel represents the straight line trajectory in the branching plane in the vicinity of the conical intersection, while the lower panel represents a vertical cut on the double cone potential highlighting the hyperbolic profile.

Although the LZ model has been derived, and used predominantly, for one-dimensional systems in the context of atomic and molecular collisions (Child, 1974; Nikitin and Umanski, 1984), it is noted that a classical trajectory is intrinsically a one dimensional geometrical object and the model here discussed is applicable to systems of any dimension where the nuclear motion is treated classically, and in particular in the vicinity of a conical intersection. Equation (74) is derived for a system tracing an hyperbolic adiabatic energy profile Equation (65) with a constant velocity [Equation (70)]. It is important to note that a straight line trajectory in the branching space in the vicinity of a conical intersection, where the potential energy surfaces have the shape of a double cone given by Equation (33), traces an hyperbolic adiabatic profile (compare Figures 4, 5) (Teller, 1937, 1969; Nikitin, 1968, 1974; Child, 1974; Malhado and Hynes, 2008). Geometrically such energy profile is the intersection of a double cone surface with a plane parallel to its axis. The LZ Equation (74) thus gives a probability of non-adiabatic transition of a straight line classical trajectory passing in the vicinity of a conical intersection (Teller, 1937, 1969; Nikitin, 1968, 1974; Child, 1974; Desouter-Lecomte et al., 1985; Alijah and Nikitin, 1999; Malhado and Hynes, 2008), under the restrictions in which it is derived. This is true even for a trajectory that passes exactly at the degeneracy point—an unlikely event given the lower dimensionality of the intersection space compared to nuclear coordinate space—when the adiabatic energy gap in Equation (74) vanishes and the probability of non-adiabatic transition is unity, as expected.

The LZ model itself has been at the center of theoretical approaches to non-adiabatic effects in atomic and molecular collision (Child, 1974; Nikitin and Umanski, 1984), and in this context has seen many extensions (Coulson and Zalewski, 1962; Bikhovskiǐ et al., 1965; Heinrichs, 1968; Delos and Thorson, 1972; Nakamura and Zhu, 1996). Nonetheless, the original LZ Equation (74) remains as a standard, with demonstrated usefulness in many applications (Tully and Preston, 1971; Heller and Brown, 1983; Lorquet and Leyh-Nihant, 1988; Nikitin, 1999; Nitzan, 2006; Kayanuma, 2007).

## 7. Surface hopping

In Section 5, the dynamics of the quantum electronic system was formally expressed as a function of an arbitrary classical nuclear coordinate $\vec{R}$(*t*). In Section 6 on the LZ model of non-adiabatic transitions, a very simple form of $\vec{R}$(*t*) was assumed, which was prescribed, i.e., not affected by the dynamics of the electronic part of the system. In general, however, it is necessary to go beyond the prescribed trajectory assumption and calculate a classical trajectory for the nuclei considering several coupled electronic states. In particular, it is necessary to take into account the influence of the electronic degrees of freedom on the dynamics of the nuclei. But the problem of dealing self-consistently with this type of quantum-classical interaction has no known solution and many different approaches have been developed in order to address it (Tully, 1998; Stock and Thoss, 2005). One of the most widely used of these is the surface hopping approach (Bjerre and Nikitin, 1967; Tully and Preston, 1971; Tully, 1990; Drukker, 1999) which has become a standard in dynamical studies of non-adiabatic molecular problems.

In the surface hopping perspective, each system is described not by a single nuclear classical trajectory but rather by an ensemble of independent trajectories. The perspective is based in part on the ideas discussed in Section 2, namely, that in regions of the nuclear configuration space where the difference between electronic eigenvalues is large, the nuclear wavefunction associated with each electronic eigenstate has its dynamics governed by the electronic energy surface of that state, and that regions of non-adiabaticity are localized in space. Accordingly, each trajectory in the surface hopping description has its motion determined by electronic adiabatic energy surfaces. Given the analogy between the coefficients 〈$\vec{R}$, ϕ_*n*_; $\vec{R}$|Ψ〉, the nuclear wavefunction, in the Born-Oppenheimer expansion Equation (7), and the electronic eigenstate expansion coefficients 〈ϕ_*n*_; $\vec{R}$|Φ; $\vec{R}$〉 in a quantum classical-description, discussed in Section 5, it is natural that the distribution of the classical trajectories among adiabatic electronic states be assigned according to the weights of the coefficients |〈ϕ_*n*_; $\vec{R}$|Φ; $\vec{R}$〉|^2^ = |*c*_*n*_|^2^. In regions where the electronic energy gap is small, these coefficients will vary according to Equation (56), and a corresponding fraction of the trajectories will switch electronic state and be propagated on a different potential energy surface. These surface switches, called hops, are instantaneous in time and taken to be vertical, i.e., the nuclear positions are conserved. Since each trajectory is independent of all the others, the hopping process when a trajectory reaches a region of non-adiabaticity is a stochastic process.

Historically, many simulations (Bjerre and Nikitin, 1967; Tully and Preston, 1971; Heller and Brown, 1983) have implemented surface hopping algorithms in which the classical trajectories are propagated on adiabatic energy surfaces until a predefined region of non-adiabaticity would be reached, at which point a stochastic decision about surface hopping would be made, usually based on a transition probability given by the LZ expression Equation (74) [or the improved Zhu-Nakamura Equations (Oloyede et al., 2006)].

In the case of non-adiabatic decay through a conical intersection—where the potential energy surfaces in the branching space coordinates are given by Equation (33) in the vicinity of the degeneracy—the nuclear dynamics of the ensemble of trajectories is initially determined by the upper cone potential energy surfaces, and the most likely trajectory point where hops may occur is the inner turning point of the radial motion (Alijah and Nikitin, 1999; Malhado and Hynes, 2008). The origin of this likelihood is not so much because this corresponds to the point along the trajectory where the energy gap between eigenstates is smallest *per se*, but rather is because the magnitude of the derivative non-adiabatic coupling vectors is maximum and collinear with the velocity which is maximum and along the tangential direction (see Figure 5); thus according to Equation (56) and Figure 3 the coupling between states will be maximum at these points. It should also be noted that a system with the special case of an overdamped trajectory, that follows the gradient toward the conical intersection in a minimum energy path, and will not be subject to any electronic coupling until it reaches the point of degeneracy (Figure 3), where the non-adiabatic coupling is singular (thus not defined). This type of trajectories, often computed and used to determine the basic mechanism of photochemical reactions, may strictly not lead to significant electronic decay, although any slight deviation that closely misses the conical intersection will (see Figure 3).

As a simulation method however, the approach described above is somewhat restrictive. This is primarily because the region of nuclear space where hops occur is defined and fixed *a priori*, rather than being based on the time propagation of the electronic coefficients through Equation (49) as a function of an arbitrary nuclear dynamics. Further, at the transition point not all the conditions under which the LZ equation was derived may be satisfied. In particular, the shape of the electronic energy profile along the trajectory may not necessarily be hyperbolic (Figure 4), the velocity need not be constant, etc., and in consequence the LZ Equation (74) may not provide a good description for the transition probability.

The current standard surface hopping simulation scheme is the Tully fewest switches algorithm (Tully, 1990; Coker, 1993; Drukker, 1999; Barbatti, 2011)—which is also called “molecular dynamics with quantum transitions”—does not suffer from the drawbacks just mentioned. Comparison of the results of simulations using the fewest switches algorithm and with full quantum dynamic simulations may range from good to fair (Müller and Stock, 1997; Topaler et al., 1997; Jasper and Truhlar, 2005). But this surface hopping approach is found to give at least good qualitative agreement in general, and the fewest switches algorithm is usually taken as the reference for comparison with other classical nuclear non-adiabatic approaches. A detail discussion of this algorithm is given next.

### 7.1. Fewest switches algorithm

In this brief description of the fewest switches algorithm, only two electronic states will be considered, but the algorithm scales efficiently to an arbitrary number of states.

In the fewest switches algorithm (Tully, 1990; Coker, 1993; Drukker, 1999; Barbatti, 2011), the time evolution both of the quantum electronic part, through Equation (49), and of the ensemble of classical trajectories is effected in parallel ^11^. In the large majority of cases each trajectory $\vec{R}$(*t*) is propagated on the adiabatic potential energy surface of one state according to inertial classical mechanics. It is however possible to use a different propagation scheme (maintaining each trajectory associated with one adiabatic surface) to include other effects, such as nuclear momentum dissipation (Cattaneo et al., 1999; Malhado et al., 2011; Nelson et al., 2011) or quantum tunneling (Shushkov et al., 2012). In terms of the electronic structure evolution, the two state system in the adiabatic representation Equation (49), upon noting Equations (50) and (54), can be written in the form of the two Equations

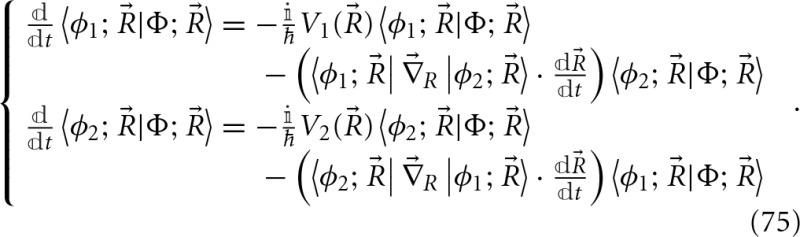



In these equations the fact has been used that for a real electronic wavefunction diagonal elements of the non-adiabatic derivative coupling matrix 〈ϕ_*n*_; $\vec{R}$|$\vec{\nabla }$_*R*_|ϕ_*n*_; $\vec{R}$〉 are equal to zero. Due to the anti-hermitian character of this matrix, one has 〈ϕ_2_; $\vec{R}$|$\vec{\nabla }$_*R*_|ϕ_1_; $\vec{R}$〉 = −〈ϕ_1_; $\vec{R}$|$\vec{\nabla }$_*R*_|ϕ_2_; $\vec{R}$〉.

At each time step, each trajectory is given the opportunity to perform a surface hop according to the time variation of the coefficients |〈ϕ_*n*_; $\vec{R}$|Φ; $\vec{R}$〉|^2^. The hopping probability of a trajectory in state 1 to state 2 at each time, *p*^1→2^_*hop*_(*t*) (often called *g*_12_ in the literature), is equal to the fractional variation of the coefficient |〈ϕ_1_; $\vec{R}$|Φ; $\vec{R}$〉|^2^ in that time step and can be written as

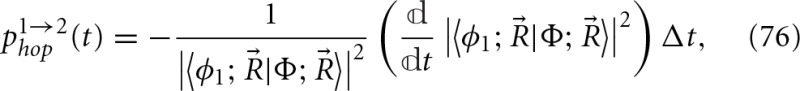



where Δ*t* is the time step length, and the minus sign indicates that a negative derivative of the electronic coefficient implies a higher probability for the trajectory to hop out of state 1. When the coefficient |〈ϕ_1_; $\vec{R}$|Φ; $\vec{R}$〉|^2^ increases, Equation (76) yields a negative value; when this occurs a zero probability of hopping is assigned and no hop out of this state is made. In this case, increase in the fraction of trajectories in the state 1 surface is achieved by hops out of state 2. Such procedure is designed to minimize the number of hops between surfaces (Tully, 1990), hence the name of *fewest switches* given to the algorithm ^12^^,^^13^.

The time derivative term in Equation (76) can be calculated, via Equation (75), from

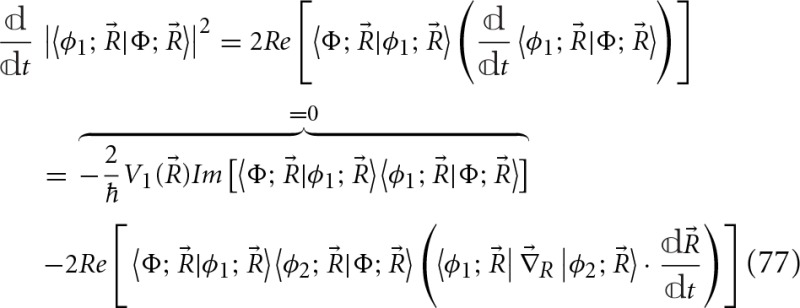



where *Re* and *Im* stand for the real and imaginary parts, respectively. The first term of the last equality in the previous equation vanishes since it involves the imaginary part of the real quantity |〈ϕ_1_; $\vec{R}$Φ; $\vec{R}$〉|^2^. With this result, the prescription for hopping probability Equation (76) is complete. Finally, by interchanging indexes in Equations (76) and (77), equations are obtained for a trajectory on the surface of state 2 to hop to the surface of state 1.

When a surface hop occurs, there is an instantaneous change in the trajectory's potential energy which is equal to the adiabatic energy gap at the point of the hop. In order to conserve total energy, the kinetic energy of the nuclei needs to change; this change is accomplished by appropriately rescaling the velocity vector. From Equation (77) it is seen that it is the component of the nuclear velocity vector along the non-adiabatic derivative coupling vector 〈ϕ_*n*_; $\vec{R}$|$\vec{\nabla }$_*R*_|ϕ_*m*_; $\vec{R}$〉 that drives the change in the quantum system, so it is this component of the velocity that is rescaled (Coker, 1993; Mei and Coker, 1996; Müller and Stock, 1997). This *ad hoc* velocity rescaling can be seen as a distribution of the electronic energy to the nuclear degrees of freedom (in the case of a hop to a lower energy electronic state) or as a collection of electronic energy in those degrees of freedom (in a case of a hop to a higher energy state).

A special case connected to the issues of velocity rescaling arises when the kinetic energy along the non-adiabatic derivative coupling vector direction is not enough to compensate for the potential energy gap, so that such a transition is classically forbidden. This occurs when a trajectory on a lower energy surface, which by comparison of a random number with the result from Equation (76), is indicated to hop to a higher energy surface. This situation is called a frustrated hop and for such a trajectory the hop is not performed. A common prescription here is that the trajectory is taken to continue on the original surface with the same velocity as before the frustrated hop attempt (Müller and Stock, 1997). The alternative prescription of inverting the velocity direction (Mei and Coker, 1996) has been shown to give negligibly different results (Spezia et al., 2006) ^14^. The existence of frustrated hops leads to the feature that the fraction of trajectories in each state is then not the same as the electronic state coefficients |〈ϕ_*n*_; $\vec{R}$|Φ; $\vec{R}$〉|^2^ (as it should), which has been considered as an inconsistency of the method (Fang and Hammes-Schiffer, 1999). Nevertheless, frustrated hops have been shown to be a necessary feature of the algorithm for a system to approach a Boltzmann distribution (Schmidt et al., 2008) in a thermalized system, a condition of proper equilibrium.

**Figure 6:**
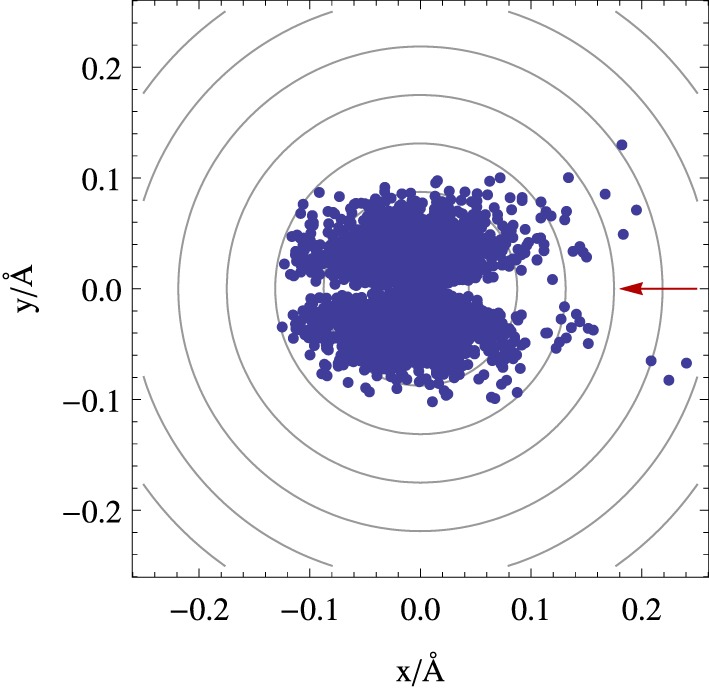
**Position where hops from the upper to the lower surface occur in a fewest switches surface hopping simulation near a conical intersection given by Equation (33)**. The initial distribution of trajectories has a gaussian distribution along the *y* axis with a standard deviation of 0.04 Å, and are started with a velocity only along *x* (see Appendix Section Simulation Details). The red arrow indicates the direction of the incoming distribution of classical trajectories and the contour lines represent energy contours for the upper surface (or the electronic eigenstate energy gap).

Figure 6 illustrates an application of the fewest switches algorithm to a conical intersection case. It shows the positions where trajectories hop from the upper to the lower surface in the vicinity of a symmetric conical intersection, for a system with an initial gaussian distribution of trajectories starting on the upper eigenstate (full details about the simulation are given in the Appendix Section Simulation Details). Figure 6 reveals a pattern reminiscent of that found in Figure 3, showing that although the potential energy surfaces, the energy gap and the derivative non-adiabatic vector field all have radial symmetry in this case, the surface hopping probability is not evenly distributed along the angular coordinate due to the approach to the conical intersection taking place from a particular direction on the branching space. In particular, there is a lower transition probability along the axis connecting the nuclear position of the system and the conical intersection it is approaching when it is collinear with the system's velocity (as can be seen by the depletion of transitions along the *x* axis in Figure 6); and example of this is the case of a minimum energy path leading to a conical intersection. Such an asymmetric pattern may have an effect on the branching ratios of the outcome of photochemical reactions, and it reinforces the importance of subtle dynamical effects which are beyond a static picture which focus solely on locating the position of conical intersections in nuclear space.

**Figure 7:**
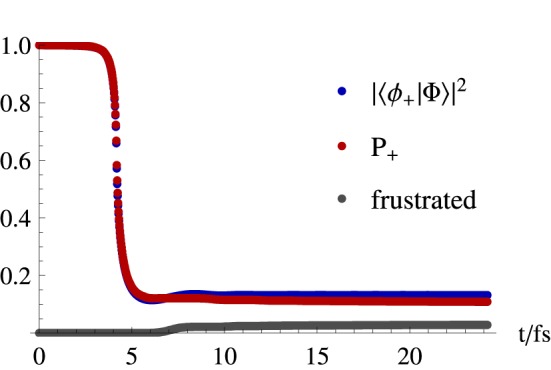
**Upper state population evolution in a fewest switches simulation of an initial gaussian distribution of trajectories in a double cone potential given by Equation (33)**. Represented are the square of the modulus of the excited electronic eigenstate component of the time dependent electronic state which is propagated in time through Equation (75), and the fraction of trajectories propagated on the upper state surface (*P*_+_). Also represented is the cumulative number of frustrated hops normalized by the total number of trajectories.

The time dependence of the hops shown in Figure 6 can be seen in Figure 7. This latter figure shows that in a system where the trajectories pass once through the conical intersection region, there is a decay of about 89% in the number of trajectories propagated on the upper state surface. It is seen that this fraction of trajectories closely follows the quantum evolution of the electronic population |ϕ_*n*_; $\vec{R}$|Φ; $\vec{R}$〉|^2^, and that frustrated hops play a marginal role, since after 24fs of simulation they correspond to about 3% of the total number of hops.

Such close agreement between fraction of trajectories propagated in one state and the quantum population is however not to be expected (Granucci and Persico, 2007) when the system crosses regions of non-adiabaticity more than once and divides into groups of trajectories exploring different regions of nuclear coordinate space (Thachuk et al., 1998). This is related to the intrinsic differences in the propagation of the quantum electronic degrees of freedom with a classical and quantum description of the nuclei, as it is detailed next.

### 7.2. Electronic quantum coherence in fewest switches surface hopping

**Figure 8:**
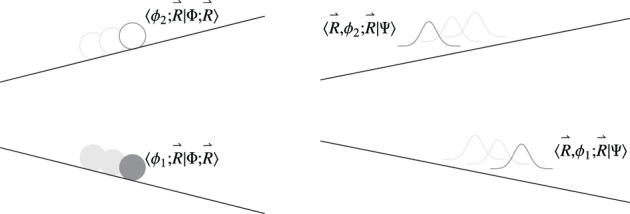
**Comparison of the evolution of the system in a surface hopping scheme with the trajectory propagated on the lower potential energy surface (left panel) and a system with quantum mechanical nuclear motion (right panel)**. It is crucial to note that in the fewest switches scheme, although the trajectory is being propagated on the lower surface (in this example), it can be seen as being followed by a “ghost” trajectory on the upper state associated with the coefficient 〈ϕ_2_; $\vec{R}$|Φ; $\vec{R}$〉. These coefficients are propagated in time through Equation (75) and in a conventional fewest switches scheme are *not* reset after a hop. Nuclear wavefunctions 〈$\vec{R}$, ϕ_1_; $\vec{R}$|Ψ〉 and 〈$\vec{R}$, ϕ_2_; $\vec{R}$|Ψ〉 associated with different states will evolve to explore different regions of nuclear position space.

Besides the obvious differences in the nuclear dynamics when changing from a quantum treatment of the nuclei to classical phase space one, this approximation also has implications in the electronic dynamics itself, beyond those already mentioned in Section 5. These differences are illustrated in Figure 8, and can be better appreciated in a density matrix formalism (Cohen-Tannoudji et al., 1977) used in the following.

A revealing way to discuss this issue is to consider the evolution of the density matrix corresponding to the electronic part of the system, which in the case classical nuclei is the full density matrix of the quantum system ρ^Φ^_*fast*_, while for a quantum description of the nuclei ρ^Ψ^_*fast*_ implies a partial trace over the nuclear degrees of freedom,

(78) $$\left\{ \begin{array}{l} \rho_{fast}^{\Psi }=Tr_{\vec{R}}(\rho^{\Psi })\text{ with }\rho^{\Psi }=|\Psi \rangle \langle \Psi |\\ \rho_{fast}^{\Phi }=|\Phi ;\vec{R}\rangle \langle \Phi ;\vec{R}n| \end{array}\right.\;.$$

The explicit form of these density matrix elements is

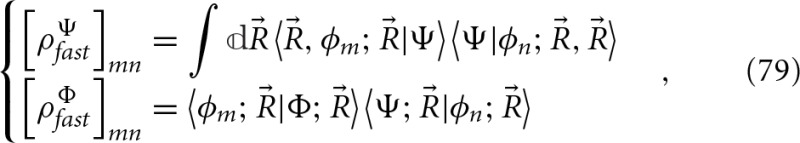



where the partial trace over quantum nuclear degrees of freedom takes the form of the integral over nuclear positions $\vec{R}$. The diagonal elements of these density matrices correspond to the populations of each electronic state, and off-diagonal terms are called electronic coherences. Important differences occur in the time evolution of these electronic coherences for a classical or quantum description of the nuclei. From the right panel of Figure 8 it is possible to see that as the nuclear wavefunctions in a two state system associated with each electronic state evolve in time, their overlap in general diminishes and the coherences ${\left[\rho_{fast}^{\Psi }\right]}_{12}$ in Equation (79) tend to zero. This decay of the off-diagonal terms of the electronic density matrix is called electronic quantum decoherence (Thachuk et al., 1998; Fiete and Heller, 2003; Miller, 2012) and the system will evolve toward a statistical (or incoherent) superposition of electronic states, described completely by the populations (Cohen-Tannoudji et al., 1977). On the other hand, by clamping the nuclei into a classical phase space description as presented in Section 5, this effect of decoherence is lost, the terms ${\left[\rho_{fast}^{\Phi }\right]}_{12}$ in Equation (79) do not tend to zero (apart from oscillations), and each trajectory evolves as a coherent superposition of electronic states ^15^.

Note that a swarm of trajectories in a surface hopping description is able to describe naturally the branching of the system into different regions of nuclear position space as each trajectory is independent. Instead, the issue is centered on the evolution of the quantum electronic degrees of freedom for each trajectory, which can lead to important qualitative differences between the same system described by a classical or quantum nuclear description (Thachuk et al., 1998; Granucci and Persico, 2007). The lack of the decoherence effect is a well-known (Tully, 1990; Prezhdo and Rossky, 1997; Zhu et al., 2004; Granucci and Persico, 2007) limitation of the fewest switches methodology as described above (and is also a limitation of many other mixed quantum-classical methods); it is a consequence of the prescription of the algorithm that trajectories be treated independently (Tully, 1998; Granucci and Persico, 2007), as to properly account for this effect the quantum electronic evolution of trajectories propagating in one state would need to depend on the positions of the trajectories on other states. Trajectory independence is nevertheless at the heart of the surface hopping approach, and while the treatment of mixed quantum-classical systems is still an open problem, several *ad hoc* mechanisms have been devised to introduce the effects of decoherence (Prezhdo and Rossky, 1997; Granucci and Persico, 2007; Granucci et al., 2010; Shenvi et al., 2011; Subotnik and Shenvi, 2011; Jaeger et al., 2012; Subotnik et al., 2013; Bajo et al., 2014), which improve the correspondence between the fraction of trajectories propagated on the surface of each state and the quantum population of that same state.

## 8. Concluding remarks

Conical intersections are confluences of potential energy surfaces where the standard Born-Oppenheimer or adiabatic approximation—which allows the familiar images and characterizations of molecular structure and many dynamic and reactive processes—breaks down. These intersections are of very considerable interest in photophysical, photochemical, and photobiological systems, since they provide regions of rapid and efficient non-adiabatic transitions between excited electronic and ground electronic states, as well as between excited states.

This review discussed a number of the key ingredients required to comprehend and describe various aspects of conical intersections, including the Born-Oppenheimer approximation itself and its breakdown, non-adiabatic transitions in a perspective where nuclear motion is treated by classical mechanics, including their description within the Landau-Zener framework, and the surface-hopping methodology to allow a classical mechanical treatment with the simultaneous handling of non-adiabatic transition dynamics between potential surfaces. This analysis highlights the importance of the combination of nuclear velocity and derivative non-adiabatic couplings to the overall dynamics of the system. Clearly, this review's treatment can only serve as an introduction to the realm of conical intersections, which promises to continue its growth with important implications for some time to come.

### Conflict of interest statement

The authors declare that the research was conducted in the absence of any commercial or financial relationships that could be construed as a potential conflict of interest.

## Footnotes

^1^The dimension of _*fast*_ is in principle infinite including the ionization continuum which has raised concerns about the formal validity of the expansion Equation (7) (Woolley and Sutcliffe, 1976; Sutcliffe and Woolley, 2012). However, for the problems involving non-ionized molecular systems it is legitimate in practice to consider only a finite number of bound states.

^2^It is possible to exactly express the total wavefunction of the system as a single term of the form of Equation (13) (Hunter, 1975; Cerderbaum, 2013), but contrary to the Born-Oppenheimer approximation, in general the function ϕ($\vec{R}$, *r*) in such a product is not an eigenfunction of the electronic Hamiltonian. The nature of the approximation is thus not the fact that only one term of the expansion is used but rather the form of this term.

^3^Terms of the form 〈ϕ_*n*_; $\vec{R}$|∇^2^_*R*_α__|ϕ_*n*_; $\vec{R}$〉 are called diagonal elements of the non-adiabatic coupling even if strictly speaking these diagonal terms do not couple different electronic states. Coupling between different electronic states is provided by off-diagonal terms in Equation (11).

^4^In the literature the terms Born-Oppenheimer and adiabatic approximations used here are often interchanged so that care in using these terms is advisable.

^5^Provided the degeneracy of electronic states does not hinder the electronic structure method chosen.

^6^An electronic basis set which is independent of the nuclear coordinates, is the crude-adiabatic basis (Longuet-Higgins, 1961) which corresponds to the eigenstates of the electronic Hamiltonian at a fixed nuclear geometry $\vec{R}$_0_ (usually the equilibrium geometry) but not for any other value of $\vec{R}$. This is however not a practical choice since, although in theory these states can form a complete basis, any small truncation will provide a bad description of the electronic states away from $\vec{R}$_0_ and a great number of states needs to be used.

^7^There are cases where the variation of the electronic Hamiltonian with the nuclear coordinates is of higher order (Carrington, 1972), a notable example being the Renner-Teller intersection involving bending modes in linear molecules. Such cases are not considered in the present study.

^8^There are some methods that use trajectories to guide evolving basis set functions in order to describe the nuclear wavefunction (Ben-Nun and Martínez, 2002; Worth et al., 2004; Shalashilin and Burghardt, 2008). These methods are not explicitly addressed in this work, where instead the Hilbert subspace _*slow*_ is replaced by a simple classical phase space description of the nuclei.

^9^With no loss of generality, but implying *t*_0_ < 0, the choice of time zero is made such that *z*(*t* = 0) = *z*_*c*_.

^10^A recently proposed alternative derivation can be found in reference (Wittig, 2005).

^11^In terms of numerical implementation, integration of Equation (49) requires a smaller time step than the evolution of the classical degrees of freedom.

^12^Since each trajectory is independent, with different histories and different classical and quantum evolutions, the fact that the coefficient |〈ϕ_1_; $\vec{R}$|Φ; $\vec{R}$|^2^ increases for a trajectory A on state 1, does not affect the probability of any other trajectory B on state 2 to hop to state 1, as this probability will depend on trajectory's B own quantum propagation. However, for a sufficiently high number of trajectories, there will be B trajectories on state 2 whose evolution mirror that of trajectory A, and a finite probability to hop from state 2 to state 1.

^13^Most surface hopping algorithms follow the so called “anteater” procedure (Tully and Preston, 1971) just described, by which upon a hop a trajectory simply resumes propagation on a new potential energy surface. An alternative procedure is the “ants” approach (Tully and Preston, 1971) where each trajectory acts like a swarm of trajectories that at a hopping event branches between the two states according to the designated hopping probability, each branch continuing independent from each other. The “ants” approach has better statistical properties but a much higher computational cost due to trajectory branching. A third scheme called “army ants” was developed (Nangia et al., 2004) to treat weak coupling systems and sample low probability events while keeping computations feasible.

^14^Another approach (Oloyede et al., 2006) to deal with frustrated hops is to make a non-vertical transition, in violation of the Franck-Condon principle, where associated to the electronic transition there is a displacement in the nuclear coordinates.

^15^On the opposing side of the spectrum of a system with a fully coherent electronic evolution, is a surface hopping scheme using a LZ type of equation to compute non-adiabatic transition probabilities. Because of condition Equation (67), the system is effectively “collapsed” to a pure electronic state after each hop, in totally decoherent propagation [coherences between two electronic states are zero unless both states are populated (Cohen-Tannoudji et al., 1977)].

## Appendices

### Equations for the evolution of nuclear wavefunctions

This appendix derives Equation (11) for the evolution of the nuclear wavefunction 〈$\vec{R}$, ϕ_*n*_; $\vec{R}$|Ψ〉 from the Schrödinger equation of the molecular system and the use of the Born-Oppenheimer expansion.

Equation (11) is obtained by first rewriting the left hand side of equation (10) using the Born-Oppenheimer expansion (7), noting from (2) and (4) the decomposition  = _*n*_ + _*e*_, and that the nuclear kinetic energy _*n*_ operates on the space of nuclear motion _*slow*_:

Further progress is made by writing the nuclear kinetic energy operator in the configuration representation, which implies a choice of the nuclear coordinate system. This choice is not unique, and here the option is made for a rectilinear coordinate system that yields a diagonal kinetic energy operator, i.e., for which the different nuclear coordinates are not kinetically coupled (Wilson et al., 1980) (for example Jacobi coordinates). The nuclear kinetic energy can thus be written as:

where the sum extends over the nuclear coordinates and *m*_α_ is an appropriate reduced mass. Other choices of coordinate system are possible and often convenient in the explicit description of the nuclear dynamics (Chapuisat et al., 1991; Meyer, 2002; Gatti and Iung, 2009), but they result in significantly more complicated expressions than the ones presented here.

Using the explicit form of the nuclear kinetic energy operator (81), Equation (80) is rewritten as

Making use of the following properties of the derivatives of delta functions (Arfken and Weber, 2005), which for any function *f*(*x*) gives for the *n* derivative of the delta function the identity

which for *n* = 1 this becomes

and further integrating over $\vec{R^{\prime }}$ in (80), that equation becomes

From this equation and (10) and noting that the eigenstates of the electronic Hamiltonian are orthogonal (〈ϕ_*n*_; $\vec{R}$|ϕ_*m*_; $\vec{R}$〉 = δ_*nm*_), Equation (11) for the evolution of the nuclear wavefunction 〈$\vec{R}$, ϕ_*n*_; $\vec{R}$|Ψ〉 is obtained.

### Simulation details

In this appendix the technical details of the fewest switches simulations presented in Section 7.1 are given.

The 2D potential energy surfaces correspond to a double cone defined by Equation (32). Taking the branching space coordinates as having dimensions of length, *A*_*x*_ = *A*_*y*_ = 0eV.Å^−1^ and *B*_*x*_ = *B*_*y*_ = 4.2eV^2^.Å^−2^. [This equivalent to taking Equation (33) with α_*x*_ = α_*y*_ = 0 and *e* = 1 and *F* = 2.1eV.Å^−1^]. The mass associated with both *x* and *y* dimensions equals 8.2 g.mol^−1^. 10000 independent trajectories were used, initially placed at *x* = 0.26Å and with a gaussian distribution in *y* centred at *y* =0Å and a standard deviation of 0.04Å. All trajectories were started with velocities *v*_*x*_ = −5.8 × 10^−2^ Å.fs^−1^ and *v*_*y*_ = 0Å.fs^−1^. Trajectories were propagated with a Velocity Verlet algorithm using a time step of Δ*t*_*c*_ = 2.4 × 10^−2^fs. A time step 25 smaller, Δ*t*_*q*_ = 1 × 10^−3^fs, was used to propagate the electronic wavefunction in Equations (75), with quantities dependent on the nuclear position and velocity linearly interpolated between classical timesteps Δ*t*_*c*_. Trajectories are allowed to hop at every Δ*t*_*q*_ according to a probability given by Equation (76).
